# The Unified AMMI-GGE (UAG) Model: A Continuous Framework for Integrating Yield Stability and Adaptability in Multi-Environment Trials

**DOI:** 10.3390/plants15121791

**Published:** 2026-06-10

**Authors:** Hristo P. Stoyanov, Nataliya Georgieva, Valentin I. Kosev, Asparuh I. Atanasov

**Affiliations:** 1 Cereals and Legumes Breeding Department, Dobrudzha Agricultural Institute—General Toshevo, Agricultural Academy—Sofia, 9521 General Toshevo, Bulgaria; 2 Breeding and Technology Department, Institute of Forage Crops—Pleven, Agricultural Academy—Sofia, 5800 Pleven, Bulgaria; imnatalia@abv.bg (N.G.); valkosev@hotmail.com (V.I.K.); 3 Department of Mechanics and Elements of Machines, Technical University of Varna, 9010 Varna, Bulgaria; asparuh.atanasov@tu-varna.bg

**Keywords:** genotype–environment interaction, UAG framework, AMMI, GGE biplot, stability analysis, triticale

## Abstract

Understanding and modeling genotype–environment interaction (GEI) remains a cornerstone of plant breeding, directly influencing cultivar recommendation and mega-environment delineation. The Unified AMMI-GGE (UAG) framework developed in this study generalizes two of the most widely used GEI models—the Additive Main effects and Multiplicative Interaction (AMMI) and the Genotype + Genotype–Environment (GGE)—into a parametric continuum governed by the tuning parameter α ∈ [0, 1]. Within a single singular-value decomposition (SVD) structure, UAG retains the inferential rigor of AMMI while recovering the decision-oriented visualization of GGE. Cross-validation experiments across multiple schemes identified intermediate α values (0.1–0.3) and rank K = 2 as optimal, minimizing root-mean-square error while maintaining interpretability. The model was empirically validated using multi-environment yield data from triticale (×*Triticosecale* Wittmack), demonstrating its capacity to classify genotypes by adaptation type and stability. At α = 0.3, genotypes G3, G5, and G8 exhibited broad adaptation, whereas G10 and G11 showed high yield potential under favorable environments. The key novelty of UAG lies in formalizing a continuous parametric path between AMMI and GGE within a single SVD structure, enabling cross-validated model selection rather than arbitrary choice between the two paradigms. The Unified AMMI-GGE Index (UAGI) provided coherent yield–stability rankings across α-values, avoiding the inconsistencies typical of separate AMMI and GGE metrics. Overall, UAG bridges interpretability and predictive validation within a single analytical framework, offering a flexible, cross-validated, and theoretically consistent tool for modern breeding programs seeking to optimize both stability and productivity under variable environments.

## 1. Introduction

Understanding and exploiting genotype–environment interaction (GEI) remains a central challenge in crop improvement. Environmental heterogeneity, combined with complex genotype responses, often obscures the identification of stable and high-yielding cultivars [1,2,3]. Since the mid-twentieth century, statistical models such as those of Finlay and Wilkinson [4] and Eberhart and Russell [5] have provided linear frameworks for assessing adaptability and stability, but they fail to capture the multidimensional nature of GEI. The advent of bilinear and multivariate models—most notably the Additive Main effects and Multiplicative Interaction (AMMI) and the Genotype + Genotype–Environment (GGE) approaches—revolutionized the interpretation of multi-environment trials [6,7,8].

The AMMI model decomposes GEI into principal interaction axes after removing the additive main effects, allowing parsimonious diagnosis of specific genotype–environment associations [9,10]. Its strength lies in interpretive precision and statistical testing of interaction components [8,11]. Conversely, the GGE biplot, by centering data on the environmental means, visualizes the combined genotype and GEI effects relevant to cultivar recommendation and mega-environment identification [12,13]. GGE’s decision-oriented visual appeal has led to its widespread adoption in cereal breeding, particularly for wheat, barley, oat, and triticale [14,15,16,17,18]. Nevertheless, AMMI and GGE have typically been applied as parallel but distinct tools, without a unified parameterization linking their inferential and visualization domains [19,20,21].

Contemporary breeding increasingly demands analytical frameworks that combine interpretability, predictive accuracy, and empirical validation. Mixed-model and factor-analytic approaches now dominate large unbalanced datasets [22,23,24], yet their complex covariance structures hinder intuitive visualization. Similarly, site-regression and factorial regression models [19,25] offer explanatory flexibility but limited communicative clarity. Despite these advances, a fundamental methodological gap persists: AMMI and GGE have invariably been applied as parallel but distinct tools, with the choice between them made arbitrarily or based on convention rather than data structure [19,20,21]. No existing framework formalizes a continuous parametric path connecting the two models within a single estimation structure, nor provides a principled cross-validated criterion for selecting the optimal degree of centering. This gap has two practical consequences for breeding programs: First, stability rankings and genotype classifications may differ depending on which model is applied, creating inconsistencies in cultivar recommendations. Second, neither model alone can be empirically validated against the other, preventing objective assessment of which representation better fits a given dataset. This methodological gap motivates a unified framework that integrates the diagnostic rigor of AMMI with the graphical decisiveness of GGE, allowing model tuning according to data structure and breeding purpose.

The Unified AMMI-GGE (UAG) framework proposed here addresses this need by introducing a continuous parameter α ∈ [0, 1] governing the relative contribution of main-effect information to the multiplicative term within a single singular-value decomposition (SVD). When α = 0, UAG collapses to AMMI, emphasizing pure interaction. When α = 1, it reproduces the classical GGE formulation, emphasizing productivity orientation and environment grouping. Intermediate α values define a smooth continuum between interpretability and decision support, enabling cross-validated optimization of both prediction and visualization (as described for AMMI and GGE by Gauch [9]; Gauch et al. [10]; Yan and Kang [12]; Yan & Frégeau-Reid [26]). Biologically, α reflects a continuum between two adaptive strategies: At α = 0, the model captures pure crossover GEI, prioritizing the identification of broadly stable genotypes. At α = 1, the full genotypic mean is incorporated into the multiplicative term, emphasizing productivity gradients and specific adaptation. Intermediate α values, therefore, represent a tunable compromise between wide and specific adaptation, with the optimal value determined empirically by the variance structure of each dataset (i.e., the relative magnitudes of G, E, and GEI effects). Such flexibility is particularly relevant for multi-environment data in cereals, where GEI is often large and stability must be balanced with yield potential [1,17].

Triticale (×*Triticosecale* Wittmack) offers a suitable case for the empirical validation of UAG due to its high genotype–environment sensitivity and the expanding breeding interest in marginal and stress-prone regions [17,27,28,29,30]. Multi-environment analyses in triticale have demonstrated the coexistence of broadly adapted and highly specific genotypes [31,32], making it an ideal test system for evaluating model generality and stability inference.

Accordingly, this study develops and validates the Unified AMMI-GGE (UAG) model, formalizing a parametric continuum between AMMI and GGE within a common SVD structure. Through cross-validation, visualization, and application to multi-environment triticale data, the research aims to (i) identify optimal α–K configurations minimizing prediction error, (ii) assess genotype adaptation and stability under varying α, and (iii) demonstrate the practical breeding implications of a unified, interpretable, and empirically tunable GEI framework.

## 2. Results

### 2.1. Theoretical Derivation of the Unified AMMI-GGE (UAG) Model

To establish a unified analytical framework for genotype-by-environment (G × E) data, the Unified AMMI-GGE (UAG) model was formulated as a continuous generalization of the classical AMMI and GGE approaches. The detailed mathematical formulation is presented below, providing the theoretical foundation for subsequent empirical evaluation using triticale multi-environment trials.

#### 2.1.1. Theoretical Background and Motivation

The response of genotypes (G) to environments (E) is classically analyzed through bilinear models that partition observed variation into additive main effects and multiplicative interaction terms. In the standard two-way layout,

$$Y_{ge}=\mu +G_{g}+E_{e}+{\left(GE\right)}_{ge}+\varepsilon_{ge},$$

where $Y_{ge}$ is the mean yield (or trait value) of genotype g in environment e, $G_{g}$ and $E_{e}$ are additive genotype and environment main effects, and ${\left(GE\right)}_{ge}$ is the interaction deviation. The residuals $\varepsilon_{ge}$ are assumed to be independent, with a mean of zero and a variance of $\sigma^{2}/w_{ge}$, where the precision weights $w_{ge}$ reflect replication and sampling precision.

Two canonical models approximate ${\left(GE\right)}_{ge}$ by low-rank bilinear forms:

1. AMMI (Additive Main effects and Multiplicative Interaction) applies double centering, removing both genotype and environment means, and performs a singular value decomposition (SVD) of the residual $Y-1_{G}{\bar{Y}}_{\cdot }^{⊤}-{\bar{Y}}_{\cdot }^{\left(E\right)}1_{E}^{⊤}$. The model captures a pure interaction structure and is statistically orthogonal to the main effects;

2. GGE (Genotype plus Genotype-by-Environment) removes only the environment means and applies SVD to $Y-1_{G}{\bar{Y}}_{\cdot }^{⊤}$, thus analyzing the combined G+GE term. The GGE biplot retains both discriminatory and predictive components of genotypic performance, but the inclusion of G in the multiplicative term sacrifices the strict interpretability of the interaction.

Both models correspond to rank-restricted approximations of distinct linear transformations of Y. The Unified AMMI-GGE (UAG) model generalizes them by constructing a “continuous one-parameter family of transformations” $R_{\alpha }$ with 0≤α≤1 that interpolates between the AMMI and GGE residual fields. This formulation allows the data to determine, by cross-validation, the optimal level of centering that minimizes prediction error or maximizes explained variation.

#### 2.1.2. Weighted Hilbert Space and Preliminaries

Let $Y\in R^{G\times E}$ denote the matrix of genotype–environment cell means, and $W=\left(w_{ge}\right)$ a matrix of nonnegative weights, with $w_{ge}>0$ for observed cells and $w_{ge}=0$ otherwise. We define the weighted Frobenius inner product and norm as:

$${\left\langle A,B\right\rangle }_{W}=\sum_{g=1}^{G}\sum_{e=1}^{E}w_{ge}A_{ge}B_{ge},\;\;∥A{∥}_{W}^{2}={\left\langle A,A\right\rangle }_{W}.$$

In the present empirical application, weights were set to $w_{ge}=1$ for all observed cells and $w_{ge}=0$ for missing cells, reflecting the balanced fully replicated design. Under uniform weights, the weighted Frobenius inner product reduces to the standard Frobenius inner product, and the WALS algorithm converges to the classical unweighted SVD solution. For unbalanced designs, $w_{ge}$ may be specified proportional to cell replication ($w_{ge}=r_{ge}$) or to the inverse of the cell-mean prediction error variance ($w_{ge}=r_{ge}/{\hat{\sigma }}_{e}^{2}$), where $r_{ge}$ denotes the number of replications in the cell $\left(g,e\right)$ and ${\hat{\sigma }}_{e}^{2}$ is the within-environment error variance estimate.

The pair $\left(R^{G\times E},\langle \cdot ,\cdot \rangle_{W}\right)$ thus forms a finite-dimensional Hilbert space, allowing orthogonal projections and linear operators to be defined under the weighted metric.

Weighted environment means are computed as:

$${\bar{Y}}_{\cdot e}=\frac{\sum_{g}w_{ge}Y_{ge}}{\sum_{g}w_{ge}},\;e=1,\ldots ,E,$$

and assembled as the row vector ${\bar{Y}}_{\cdot }^{⊤}\in R^{E}$. Similarly, after centering by environments, the weighted genotype means are:

$${\bar{Y}}_{g\cdot }^{\left(E\right)}=\frac{\sum_{e}w_{ge}\left(Y_{ge}-{\bar{Y}}_{\cdot e}\right)}{\sum_{e}w_{ge}},\;g=1,\ldots ,G,$$

collected as the column vector ${\bar{Y}}_{\cdot }^{\left(E\right)}\in R^{G}$.

#### 2.1.3. Definition of the UAG Transformation

The UAG transformation defines the “centered field” $R_{\alpha }$ as:

$$R_{\alpha }=Y-1_{G}{\bar{Y}}_{\cdot }^{⊤}-\left(1-\alpha \right)\;{\bar{Y}}_{\cdot }^{\left(E\right)}1_{E}^{⊤},\;\alpha \in \left[0,1\right].$$

This single formula yields both limiting cases:

$$R_{0}=Y-1_{G}{\bar{Y}}_{\cdot }^{⊤}-{\bar{Y}}_{\cdot }^{\left(E\right)}1_{E}^{⊤},\;\left(\mathrm{AMMI}\;\mathrm{field}\right),$$

$$R_{1}=Y-1_{G}{\bar{Y}}_{\cdot }^{⊤},\;\left(\mathrm{GGE}\;\mathrm{field}\right).$$

Hence, $R_{\alpha }$ provides a continuous affine path between the pure-interaction (double-centered) field $R_{0}$ and the G+GE field $R_{1}$. In operator notation,

$$R_{\alpha }=C_{E}\left(Y\right)-\left(1-\alpha \right)P_{G}\left(C_{E}\left(Y\right)\right),$$

where $C_{E}$ is the projection that removes the environment means:

$$C_{E}\left(Y\right)=Y-1_{G}{\bar{Y}}_{\cdot }^{⊤},$$

and $P_{G}$ is the projection onto the space spanned by genotype means after environment centering:

$$P_{G}\left(Z\right)={\bar{Z}}_{\cdot }^{\left(E\right)}1_{E}^{⊤},\;Z=C_{E}\left(Y\right).$$

The parameter $\left(1-\alpha \right)$ thus determines the strength with which genotype averages are reintroduced into the field analyzed by the SVD.

#### 2.1.4. Weighted Low-Rank Approximation Problem

For fixed α, the model seeks a rank-K bilinear approximation to $R_{\alpha }$ minimizing the weighted residual sum of squares:

$$\left(\hat{A},\hat{B}\right)=arg\underset{A\in R^{G\times K},\;B\in R^{E\times K}}{min}f\left(A,B\right)=arg\underset{A,B}{min}\sum_{g=1}^{G}\sum_{e=1}^{E}w_{ge}{\left(R_{\alpha ,ge}-A_{g\cdot }B_{e\cdot }^{⊤}\right)}^{2}.$$

The objective can be rewritten as a quadratic form:

$$f\left(A,B\right)=∥R_{\alpha }-AB^{⊤}{∥}_{W}^{2}=∥R_{\alpha }{∥}_{W}^{2}-2\langle R_{\alpha },AB^{⊤}\rangle_{W}+∥AB^{⊤}{∥}_{W}^{2}.$$

Given B, minimizing over A yields the normal equations:

$$A_{g\cdot }^{⊤}={\left(B^{⊤}W_{g}B\right)}^{-1}B^{⊤}W_{g}R_{g\cdot }^{⊤},\;\;W_{g}=diag\left(w_{g1},\ldots ,w_{gE}\right).$$

Similarly, for fixed A,

$$B_{e\cdot }^{⊤}={\left(A^{⊤}W^{e}A\right)}^{-1}A^{⊤}W^{e}R_{\cdot e},\;\;W^{e}=diag\left(w_{1e},\ldots ,w_{Ge}\right).$$

Alternating these updates defines the “Weighted Alternating Least Squares (WALS)” algorithm, which converges monotonically to a stationary point of $f\left(A,B\right)$. Under full-rank and positive-weight conditions, local convexity guarantees the uniqueness of the factor pair up to a rotation of the latent subspace.

To obtain rotation-invariant scores, one performs a symmetric SVD on the fitted interaction:

$$\hat{M}=\hat{A}{\hat{B}}^{⊤}=U\Sigma V^{⊤},\;U^{⊤}U=V^{⊤}V=I_{K}.$$

where ***I****_K_* denotes the K × K identity matrix.

The biplot coordinates are then defined as:

$$G_{scores}=U\Sigma^{1/2},\;E_{scores}=V\Sigma^{1/2}.$$

The reconstructed mean matrix in the original scale is:

$${\hat{Y}}_{\alpha ,K}=1_{G}{\bar{Y}}_{\cdot }^{⊤}+\left(1-\alpha \right){\bar{Y}}_{\cdot }^{\left(E\right)}1_{E}^{⊤}+U\Sigma V^{⊤}.$$

The weighted proportion of variation explained in the transformed field is:

$$R_{R}^{2}\left(\alpha ,K\right)=\frac{∥\hat{M}{∥}_{W}^{2}}{∥R_{\alpha }{∥}_{W}^{2}}.$$

where $R_{R}^{2}\left(\alpha ,K\right)$ denotes the weighted coefficient of determination of the rank-K approximation in the α-transformed field.

#### 2.1.5. Model Selection and Cross-Validation

The parameters α and K were estimated empirically by minimizing cross-validated prediction error in both the original yield space (Y) and the α-transformed space ($Y_{\alpha }$). For each cross-validation scheme, two error surfaces were computed—one based on direct yield predictions and one based on the α-weighted representation—allowing for simultaneous evaluation of the model regularization and predictive efficiency. Formulas were derived analytically for each validation scheme, following the general cross-validation framework of Stone [33] and Geisser [34].

The general form of the cross-validated mean squared error (CV-MSE) is given by:

$${CV‐MSE}^{\left(S\right)}\left(\alpha ,K\right)=\frac{1}{\left|V\right|}\sum_{\left(g,e\right)\in V}w_{ge}{\left({\hat{Y}}_{\left(\alpha ,K;ge\right)}^{\left(S,-V\right)}-Y_{ge}^{\left(S\right)}\right)}^{2},$$

where $S\in \{Y,Y_{\alpha }\}$ denotes the evaluated data space, ${\hat{Y}}_{\left(\alpha ,K;ge\right)}^{\left(S,-V\right)}$ is the model prediction obtained after excluding the validation subset V, and $w_{ge}$ represents optional observation weights. The optimal parameter pair $\left(\alpha *,K*\right)$ minimizes this criterion jointly across both spaces. The superscripts −e, −g, −(g,e), and −(g,−e) indicate that the corresponding environment, genotype, cell, or genotype–environment pair has been excluded from model fitting.

The Leave-One-Environment-Out (LOEO) is:

$${CV‐MSE}_{LOEO}^{\left(S\right)}\left(\alpha ,K\right)=\frac{1}{E}\sum_{e=1}^{E}\frac{\sum_{g}w_{ge}{\left({\hat{Y}}_{\left(\alpha ,K;ge\right)}^{\left(S,-e\right)}-Y_{ge}^{\left(S\right)}\right)}^{2}}{\sum_{g}w_{ge}}.$$

Each environment e is sequentially removed from model fitting, and all genotype means for that environment are predicted. This design measures the ability of the UAG model to extrapolate genotype performance to untested sites.

The Leave-One-Genotype-Out (LOGO) is:

$${CV‐MSE}_{LOGO}^{\left(S\right)}\left(\alpha ,K\right)=\frac{1}{G}\sum_{g=1}^{G}\frac{\sum_{e}w_{ge}{\left({\hat{Y}}_{\left(\alpha ,K;ge\right)}^{\left(S,-g\right)}-Y_{ge}^{\left(S\right)}\right)}^{2}}{\sum_{e}w_{ge}}.$$

Here, each genotype g is excluded across all environments, and its performance is predicted from the remaining G−1 genotypes. This quantifies model generalization across the genotype dimension.

The Leave-One-Combination-Out (LOCO) is:

$${CV‐MSE}_{LOCO}^{\left(S\right)}\left(\alpha ,K\right)=\frac{1}{GE}\sum_{g=1}^{G}\sum_{e=1}^{E}w_{ge}{\left({\hat{Y}}_{\left(\alpha ,K;ge\right)}^{\left(S,-ge\right)}-Y_{ge}^{\left(S\right)}\right)}^{2}.$$

In this most granular validation, each individual observation $\left(g,e\right)$ is left out in turn, and its value is predicted independently. This design provides the strictest internal consistency test and is sensitive to local overfitting.

The Two-Way Leave-One-Out (LOO) is:

$${CV‐MSE}_{Two‐way}^{\left(S\right)}\left(\alpha ,K\right)=\frac{1}{GE}\sum_{g=1}^{G}\sum_{e=1}^{E}w_{ge}{\left({\hat{Y}}_{\left(\alpha ,K;ge\right)}^{\left(S,-g,-e\right)}-Y_{ge}^{\left(S\right)}\right)}^{2}.$$

This joint scheme simultaneously excludes a genotype g and an environment e in each iteration, offering an integrated measure of model generalization across both biological dimensions.

All four validation schemes were computed in parallel for both Y and $Y_{\alpha }$. The resulting surfaces of ${CV‐MSE}_{Y}\left(\alpha ,K\right)$ and ${CV‐MSE}_{Y_{\alpha }}\left(\alpha ,K\right)$ were compared to determine the most stable and generalizable configuration. In the present balanced dataset, CV-MSE values in the α-transformed space (Yα) were equivalent to those in the original yield space (Y), consistent with the fully replicated experimental structure. In unbalanced designs with missing cells, the two spaces are expected to diverge, with Yα providing improved regularization. This dual-space validation ensures that the final parameterization reflects both raw predictive capacity and the biologically interpretable structure captured through *α*-weighting.

From the CV-MSE criterion, two derived predictive error statistics were computed for each validation scheme and parameter configuration. The Prediction Residual Sum of Squares (PRESS) aggregates the weighted squared prediction error across all validation observations:

$${PRESS}^{\left(S\right)}\left(\alpha ,K\right)=\sum_{\left(g,e\right)\in V}w_{ge}{\left({\hat{Y}}_{\alpha ,K;ge}^{\left(S,-V\right)}-Y_{ge}^{\left(S\right)}\right)}^{2}$$

The root mean square error (RMSE) expresses the prediction error on the original response scale:

$${RMSE}^{\left(S\right)}\left(\alpha ,K\right)=\sqrt{\frac{{PRESS}^{\left(S\right)}\left(\alpha ,K\right)}{\sum_{\left(g,e\right)\in V}w_{ge}}}$$

For the present balanced design ($w_{ge}=1$ for all observed cells), these reduce to:

$${PRESS}^{\left(S\right)}\left(\alpha ,K\right)=\left|V\right|\cdot {CV‐MSE}^{\left(S\right)}\left(\alpha ,K\right)$$

and

$${RMSE}^{\left(S\right)}\left(\alpha ,K\right)=\sqrt{{CV‐MSE}^{\left(S\right)}\left(\alpha ,K\right)}$$

Both statistics were computed in parallel for $S\in \{Y,Y_{\alpha }\}$, yielding complementary error surfaces for each validation scheme.

#### 2.1.6. Geometric Interpretation: Mean-Stability Analysis

Let $E_{scores}^{\left(2\right)}$ and $G_{scores}^{\left(2\right)}$ denote the first two columns of the environment and genotype score matrices. The “average environment coordination” (AEC) direction is defined as

$$m_{E}=\frac{1}{E}E_{scores}^{\left(2\right)⊤}1_{E},\;v_{AEC}=\frac{m_{E}}{∥m_{E}∥}.$$

where the superscript (2) denotes the submatrix formed by the first two columns of dimensions E × 2 and G × 2, respectively

For genotype g, the projection onto $v_{AEC}$ is:

$$x_{g}=v_{AEC}^{⊤}G_{scores}^{\left(2\right)}\left(g,\cdot \right)$$

where it represents its mean performance, and the perpendicular deviation, where the subscript (g,⋅) denotes the g-th row vector of the genotype score matrix

$$y_{g}=∥G_{scores}^{\left(2\right)}\left(g,\cdot \right)-x_{g}v_{AEC}∥$$

quantifies instability. The scalar

$${\text{UAG\_IPCA\_stability}}_{g}=∥G_{scores}^{\left(2\right)}\left(g,\cdot \right)∥$$

serves as a direct stability measure, reducing to the AMMI interaction principal component amplitude when α=0. A unified selection index combining productivity and stability can be expressed as

$${UAGI}_{g}\left(w\right)=w\;z\left({\bar{Y}}_{g}\right)-\left(1-w\right)\;z\left({\text{UAG\_IPCA\_stability}}_{g}\right),$$

where $z\left(\cdot \right)$ denotes standardization and $w\in \left[0,1\right]$ controls the emphasis on yield versus stability.

#### 2.1.7. Boundary Behavior and Regularization Path

The transformation $R_{\alpha }$ defines an affine path in the space of weighted matrices. Differentiating with respect to α,

$$\frac{\partial R_{\alpha }}{\partial \alpha }={\bar{Y}}_{\cdot }^{\left(E\right)}1_{E}^{⊤}.$$

Hence, the trajectory $\alpha \mapsto R_{\alpha }$ is linear in α. The corresponding low-rank approximation problem defines a “regularization path” analogous to ridge regression: increasing α reduces the bias induced by over-centering genotypes, while decreasing α yields a “cleaner” interaction but a potentially higher variance.

The optimal value α*, minimizing cross-validated MSE, can lie anywhere in $\left[0,1\right]$. A boundary solution of α*=1 (pure GGE) indicates that the data support retaining full genotypic variation in the multiplicative kernel—typical when environmental variance dominates or the replication noise inflates main-effect estimates. Conversely, interior values 0<α*<1 occur when double centering captures a structure not explained by environment means alone. Thus, boundary optimality is not a contradiction but a legitimate outcome of the data-driven bias-variance equilibrium inherent in UAG.

Mathematically, the continuity of the SVD under smooth perturbations of $R_{\alpha }$ ensures that $U\left(\alpha \right),V\left(\alpha \right)$, and $\Sigma \left(\alpha \right)$ vary continuously for all α except at eigenvalue crossings, guaranteeing consistent geometry along the centering path.

#### 2.1.8. Statistical and Computational Properties

The UAG objective is separately convex in A and B, enabling efficient alternating updates. Each ALS iteration solves G genotype-wise and E environment-wise weighted normal systems of size K, leading to computational complexity $O\left(\left(G+E\right)K^{3}\right)$ per iteration. The fitted matrix $\hat{M}=\hat{A}{\hat{B}}^{⊤}$ is unique up to orthogonal rotations; the symmetric SVD enforces a canonical orientation.

Decomposition of weighted sums of squares yields:

$${∥Y-{\hat{Y}}_{\alpha ,K}∥}_{W}^{2}=∥R_{\alpha }-\hat{M}{∥}_{W}^{2}+∥\left(I-C_{E}\right)Y{∥}_{W}^{2}+∥\left(1-\alpha \right)P_{G}\left(C_{E}\left(Y\right)\right){∥}_{W}^{2},$$

where the last two terms represent residual main effects and centering remainders. Missing data are handled by setting $w_{ge}=0$, and **I** is the identity operator on ℝ^{G×E}^. Regularization of normal equations with ridge penalties $\lambda I_{K}$ guarantees numerical stability in unbalanced designs without altering the fitted values asymptotically.

Under standard conditions (bounded weights, fixed rank K), $\hat{M}$ is consistent for the best weighted rank-K approximation of $R_{\alpha }$ in the Hilbert-space sense.

The framework connects naturally to mixed-model factor-analytic structures: replacing Y with BLUP predictions and W with inverse prediction-error variances yields a low-rank approximation equivalent to the estimated factor-analytic covariance component.

Model fitting was performed using the Weighted Alternating Least Squares (WALS) algorithm described in Section 2.1.4, with convergence declared when the relative change in the objective function between successive iterations fell below $10^{-6}$. Numerical stability under near-singular configurations was ensured by adding a ridge penalty $\lambda =10^{-4}$ to the diagonal of the normal equations. The significance of individual interaction principal components was assessed using the F-test of Gollob [35], adapted for weighted bilinear models, with degrees of freedom for component k computed as $df_{k}=(G-1)+(E-1)-(2k-1)$. The proportion of weighted GEI variance explained by the rank-*K* approximation was quantified by $RR^{2}\left(\alpha ,K\right)$, as defined in Section 2.1.4. All computations were implemented in Python 3.11, as described in Section 4.6.

#### 2.1.9. Analytical Implications and Theoretical Unification

The UAG model unifies AMMI and GGE within a single analytical geometry:

$$\underset{\alpha \to 0}{lim}R_{\alpha }=R_{AMMI},\;\;\underset{\alpha \to 1}{lim}R_{\alpha }=R_{GGE}.$$

Because $R_{\alpha }$ is linear in α, both limiting forms and all intermediate states reside in the same affine subspace of $R^{G\times E}$. Consequently, all classical interpretive tools—interaction principal component analysis, mean–stability biplots, which–won–where polygons, and AEC projections—are defined consistently across the continuum.

From a statistical learning perspective, UAG implements a ‘centering regularization’ governed by α. It controls the degree of bias toward pure-interaction representation versus predictive generalization, with α=0 minimizing confounding and α=1 minimizing prediction error under large environmental variance. Thus, UAG forms a continuous bias–variance bridge between the two established paradigms.

The result is a theoretically complete, empirically tunable model family:

$${\hat{Y}}_{\alpha ,K}=1_{G}{\bar{Y}}_{\cdot }^{⊤}+\left(1-\alpha \right){\bar{Y}}_{\cdot }^{\left(E\right)}1_{E}^{⊤}+\sum_{k=1}^{K}\lambda_{k}\;u_{k}v_{k}^{⊤},$$

where $\left(\lambda_{k},u_{k},v_{k}\right)$ are the singular triplets of $R_{\alpha }$ in the weighted space. This expression encapsulates AMMI (α=0) and GGE (α=1) as exact special cases, while for intermediate ones, α provides an optimal linear combination of their defining features. *λ_k_* is the k-th singular value (the diagonal elements of **Σ**), and ***u****_k_* ∈ ℝ^G^ and ***v****_k_* ∈ ℝ^E^ are the corresponding left and right singular vectors of *R_α_*.

### 2.2. Empirical Evaluation of the UAG Model

The practical performance of the UAG framework was assessed using multi-environment triticale trials conducted between 2022 and 2024. Six configurations were evaluated—five with K = 2 (α = 0.0–1.0) and one with K = 3 (α = 0.3)—using symmetric scaling. The dataset exhibited highly significant genotype (G), environment (E), and G × E effects (*p* < 0.001). Intermediate α values, particularly α = 0.3, yielded the most balanced and interpretable representation of yield performance and stability, forming the empirical basis for subsequent analyses.

#### 2.2.1. Variance Partitioning and Model Performance

The partitioning of total phenotypic variation was first assessed through a classical two-way ANOVA with replication and, subsequently, through the UAG decomposition applied to the α-transformed matrices. This dual approach enabled comparison between the traditional additive partitioning of AMMI and the α-weighted unified structure of UAG.

Table 1 summarizes the ANOVA of the studied triticale genotypes (2022–2024). The environment effect was highly significant (*F* = 144.06; *p* < 0.001) and accounted for 77.20% of the total sum of squares (SS = 6,548,325.08). Genotypic differences were significant (*F* = 2.54; *p* = 0.015), explaining 10.20% of the total variation (SS = 865,432.58). The genotype-by-environment (G × E) interaction was also highly significant (*F* = 11.29; *p* < 0.001) and contributed 8.04% (SS = 681,839.79). The residual variance represented 4.56% of the total (SS = 386,445.43), confirming high experimental precision. This variance structure follows the typical pattern for replicated multi-environment trials, where environmental effects dominate but the G × E term remains decisive for cultivar adaptability and stability.

The subsequent UAG decomposition (Table 2) refined this structure by projecting each α-transformed matrix (Yα) into orthogonal multiplicative components. At α = 0.0 (AMMI2), the first two principal components (PC1 and PC2) explained 86.47% and 13.53%, respectively, jointly capturing the entire modeled interaction variance (SS = 136,367.96). At α = 0.1, PC1 and PC2 accounted for 85.89% and 13.48% (99.37% in total; SS = 138,098.82), while at α = 0.3, the respective shares were 82.03% and 13.58% (95.61%; SS = 151,945.74), with a residual sum of squares of 6677.67. Increasing the model rank to K = 3 at the same α raised the explained variance to 100%, confirming that the third component contributed only marginal information.

At higher α levels (0.9–1.0, corresponding to GGE-type structures), the proportion of variance explained by PC1–2 decreased slightly because environmental centering inflates the total variance. For α = 0.9, PC1 and PC2 captured 76.52% and 18.25% (94.77%; SS = 276,568.04) and, for α = 1.0 (GGE2), 77.75% and 17.52% (95.27%; SS = 309 454.47). Despite this decline, both components remained statistically significant (*p* < 0.001) across all α configurations, confirming that both AMMI- and GGE-like extremes describe genuine G × E structure.

Formal F-tests based on pooled mean squares (Table 2) demonstrated that PC1 and PC2 were highly significant in every α–K setting. The configuration α = 0.3, K = 2 yielded the most favorable balance between explanatory power (95.61%) and residual mean square (MSres = 238.49), excluding degenerate solutions at α = 0.0 and the partially saturated configuration at α = 0.1 (MSres = 30.85).

From a breeding perspective, the UAG model effectively concentrates systematic interaction within a limited number of components, thereby enhancing interpretability and analytical precision. Low α values emphasize stability-oriented (AMMI-like) patterns, intermediate α ≈ 0.3 provides the best compromise between yield and stability, and high α (≥0.9) highlights productivity and mega-environment delineation.

Overall, the combined ANOVA (Table 1) and UAG decomposition (Table 2) confirm a well-structured dataset with statistically significant and biologically interpretable G × E effects captured primarily by the first two UAG components.

#### 2.2.2. UAG Biplot Patterns and Genotype Clustering

**Figure 1:**
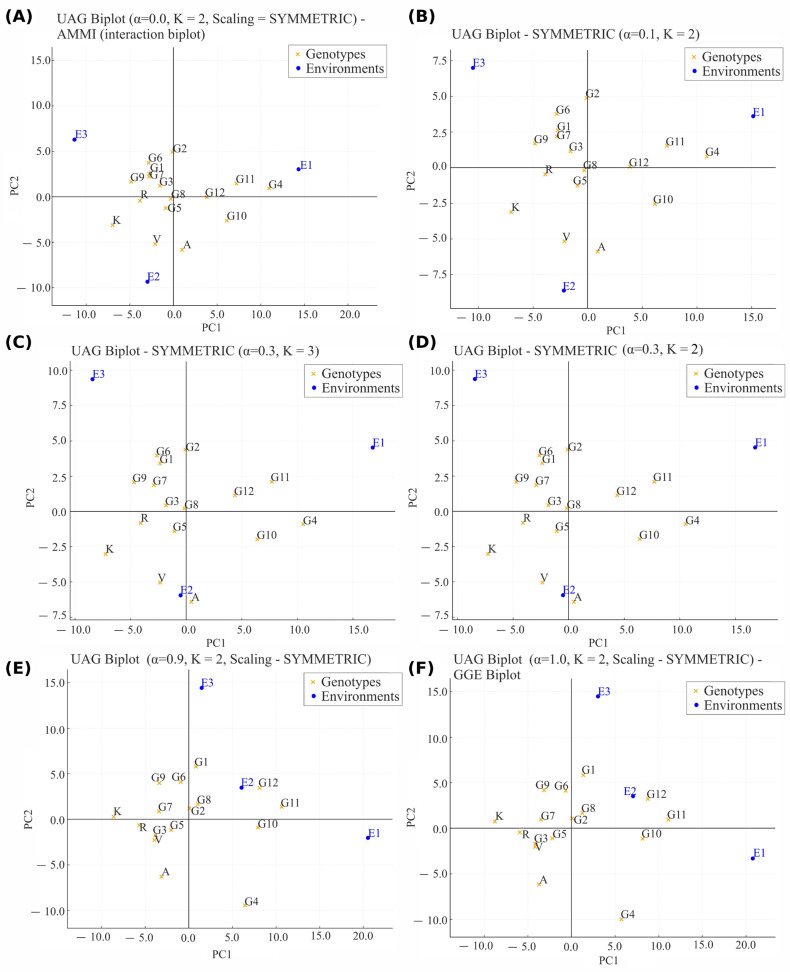
UAG biplots of the studied genotypes with different alpha (α) and K-values. (**A**) α = 0.0, K = 2; (**B**) α = 0.1, K = 2; (**C**) α = 0.3, K = 3; (**D**) α = 0.3, K = 2; (**E**) α = 0.9, K = 2; (**F**) α = 1.0, K = 2.

The biplots derived from the Unified AMMI-GGE (UAG) model (Figure 1) provided an integrative and geometrically coherent representation of the genotype–environment interaction (G × E) structure across the α continuum (0.0–1.0) under symmetric scaling.

The joint ordination of genotypic and environmental scores on the first two interaction principal components (PC1 and PC2) delineated the dominant multiplicative patterns within the dataset, offering a direct visualization of how the relative contribution of additive and interactive effects changes along the AMMI-GGE continuum.

In this configuration, the absolute magnitude of PC1 and PC2 coordinates quantifies the intensity of genotype responsiveness, while proximity to the origin indicates a more stable, environment-insensitive behavior.

At α = 0.0 (AMMI2), the ordination displayed an approximately isotropic geometry, characteristic of purely interactive models where the additive main effects are fully removed.

The genotypes were symmetrically distributed around the biplot center, reflecting balanced interaction dispersion without the dominance of any specific environment. Several lines, notably G8, G5, and G3, clustered close to the origin (|PC| ≤ 2), indicating low interaction and high general adaptability. By contrast, G4, G10, and G11 occupied distal positions along the first axis (|PC1| = 6–11), expressing pronounced environment-specific responses. Among the local check varieties, Rakita (R) was positioned relatively centrally (|PC| ≈ 3.9), while AD-7291 (A) and Kolorit (K) exhibited large interaction amplitudes (5.9–7.6), suggesting considerable environmental sensitivity. This pattern contradicts the expectation of complete neutrality among checks and confirms that some reference varieties contribute substantially to the interaction term, thereby enhancing the discriminative potential of the model.

At α = 0.1, the general structure remained similar but with a subtle elongation along PC1, indicating that a small fraction of environmental main effects began to influence the multiplicative term. The relative positions of the genotypes were largely conserved, with G8 maintaining the lowest interaction amplitude (|PC| ≈ 0.4), confirming its exceptional stability.

Lines G3 and G5 remained near the origin, while G4, G10, and G11 continued to express high responsiveness to favorable conditions. The check varieties retained their relative ranking in interaction magnitude, with R the least and A the most variable.

At α = 0.3 (K = 2), the UAG ordination reached its highest interpretive resolution. The inclusion of a moderate portion of environmental main effects increased the separation along PC1, resulting in a clear differentiation between stable and responsive genotypes. G3, G5, and G8 remained close to the origin, reflecting consistent performance across test sites and minimal interaction deviation. Conversely, G4, G10, G11, and G12 showed large loadings on both PC1 and PC2 (|PC| ≈ 6–8), indicating strong positive associations with high-yielding environments.

G4, with the highest overall amplitude (|PC| ≈ 10.6), exhibited the most unstable pattern, suggesting a highly environment-dependent response. Among the checks, A and K remained distinctly peripheral, while R was again positioned closest to the center, confirming its relatively balanced behavior.

When the model rank was increased to K = 3 at the same α level, the spatial configuration changed only marginally, showing that the essential G × E structure is already captured by the first two components.

At higher α values (0.9 and 1.0, corresponding to GGE-type configurations), the biplots became more elongated and environment-oriented. The first axis dominated the variation, integrating the influence of mean yield into the interaction structure. Genotypes G2, G8, and G5 retained near-central positions (|PC| < 4), maintaining balanced adaptability, while G10, G11, G12, and G4 remained at the outer margins, expressing pronounced environmental responsiveness. The check varieties continued to show moderate-to-high interaction magnitudes (|PC| = 5–8), confirming that they cannot be regarded as stability benchmarks but rather as comparative standards defining the interaction range.

**Figure 2:**
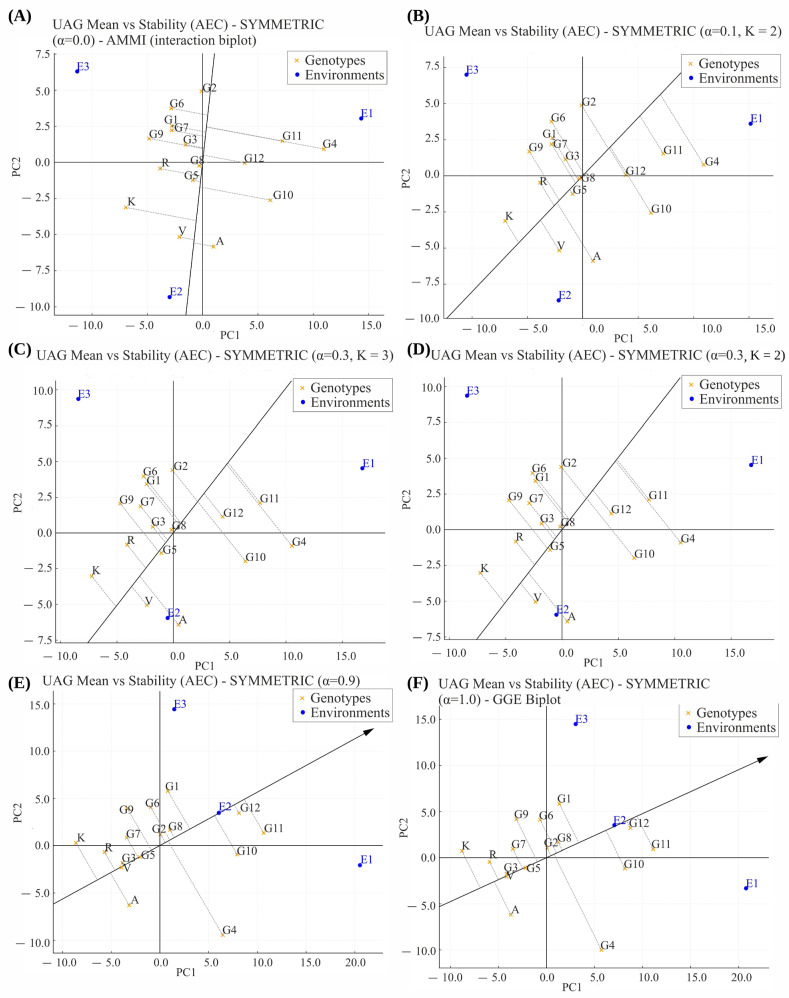
Mean vs. stability biplot for different UAG models (**A**) α = 0.0, K = 2; (**B**) α = 0.1, K = 2; (**C**) α = 0.3, K = 3; (**D**) α = 0.3, K = 2; (**E**) α = 0.9, K = 2; (**F**) α = 1.0, K = 2.

Figure 2 presents the mean vs. stability (AEC) biplots derived from the same configurations. The vector of the average environment (AEC abscissa) represents the direction of increasing mean yield, while the projection distances from this axis quantify instability. In this representation, G10 and G11 were aligned with the positive direction of the yield vector, indicating high productivity combined with moderate to low stability. G3, G5, and G12 occupied intermediate positions, expressing balanced adaptability and consistent performance across environments.

G2 and G8 were located near the origin, confirming their stable, yet average-yielding, character.

The check varieties (A, V, K, R) clustered outside the central sectors of the biplots, reflecting their role as reference points for assessing genotype dispersion rather than as models of stability.

Across the α continuum, the UAG biplots clearly delineated three major interpretive domains:

(1) Stable and broadly adapted genotypes—G5, and particularly G8, which maintained minimal PC1-PC2 deviations in all configurations;

(2) High-yield, environment-responsive genotypes—G10, G11, G12, and G4, characterized by large absolute PC loadings and strong alignment with high-yield environments;

(3) Reference check varieties—A, V, R, and K, which, despite variable interaction amplitudes, consistently provided an internal comparative baseline.

The gradual transformation of biplot geometry from α = 0.0 to α = 1.0 demonstrates the analytical flexibility of the UAG framework: lower α values emphasize stability and balanced G × E effects, whereas higher α values highlight productivity gradients and environment-specific responses. This continuum provides a unified, quantitative basis for interpreting both stability and responsiveness patterns in triticale genotypes, ensuring coherence between graphical and analytical assessments within multi-environment testing programs.

#### 2.2.3. Stability and Unified Selection Indices

The evaluation of genotype performance within the Unified AMMI-GGE (UAG) framework integrates two complementary analytical dimensions: the UAG_IPCA_Stability, which quantifies the magnitude of genotype-by-environment interaction (G × E), and the Unified AMMI-GGE Index (UAGI), which combines stability and productivity into a single selection criterion. Together, these parameters provide a coherent picture of adaptability and yield potential across the α continuum.

The UAG_IPCA_Stability parameter expresses the Euclidean distance of each genotype from the origin in the α-transformed interaction space, effectively summarizing the magnitude of its interactive deviation. Smaller values denote consistent performance across environments (wide adaptability), whereas large values indicate high responsiveness or instability. Because the metric is computed directly from the same α-dependent decomposition used in the biplots, it provides a numerical analogue of their geometric interpretation.

Across all configurations (α = 0.0, 0.1, 0.3, 0.9, 1.0, and α = 0.3 with K = 3), the stability range varied from 0.28 to 11.5 across all α configurations, with the maximum values occurring at higher α levels (0.9–1.0) where yield-associated variance inflates the interaction scores, confirming the presence of both highly stable and highly sensitive genotypes in the population. At α = 0.0 (AMMI), values ranged from 0.40 (G8) to 10.99 (G4), with an average of 4.86. The same pattern persisted at α = 0.1 (0.36–10.94) and α = 0.3 (0.28–10.56), with G8 consistently showing the lowest instability scores. These results identify G8 as the most stable genotype in the dataset, exhibiting minimal G × E variation regardless of α. Several other genotypes (G1, G2, G6, and G12) maintained intermediate stability (3–5), typical of broadly adapted material.

Conversely, G4 was the most unstable across all configurations, exceeding 10.0 in every model, indicating strong crossover interactions and environment-specific adaptation. Among the check varieties, Rakita (R) and Vihren (V) exhibited moderate stability (4–6), whereas Kolorit (K) and AD-7291 (A) were less stable, with values near the upper quartile of the distribution.

When α increased to 0.9 and 1.0, the inclusion of yield-related variance elevated overall instability (mean ≈ 6.0). The stability hierarchy partially shifted: G2 emerged as the most stable line (UAG_IPCA_Stability = 1.1–1.2), while G8 remained within the top quartile but with slightly increased variability. This transition reflects the theoretical behavior of the UAG model, where higher α values amplify yield-associated differences, thus reducing apparent stability. Nonetheless, the internal consistency of genotype rankings demonstrates that UAG_IPCA_Stability is a robust indicator of interaction behavior across the AMMI-GGE continuum.

While stability alone provides critical insight into genotype reliability, practical breeding decisions require a simultaneous consideration of yield performance. For this purpose, the Unified AMMI-GGE Index (UAGI) was computed.

At α = 0.0 (AMMI), where stability dominates, the highest UAGI values corresponded to genotypes with low stability and high yields—G11, G12, and G8. The check variety Kolorit (K) also ranked high, supporting its interpretation as a performance benchmark under interaction-focused models. At α = 0.3 (K = 2), the index reached its most balanced expression. Here, G8 maintained the top position, combining low instability (0.28) with above-average yield, followed closely by G12 and G6, both displaying favorable trade-offs between productivity and resilience. Increasing the model rank to K = 3 at the same α did not change the ranking, confirming that the two-component UAG model sufficiently captures the relevant structure. At higher α values (0.9 and 1.0), the UAGI values increasingly reflected the yield potential. Genotypes G10 and G12 rose to dominance due to their high productivity, while G8 retained a competitive position because of its inherent stability. Meanwhile, G4 and K, characterized by high instability, consistently recorded the lowest UAGI values.

**Figure 3:**
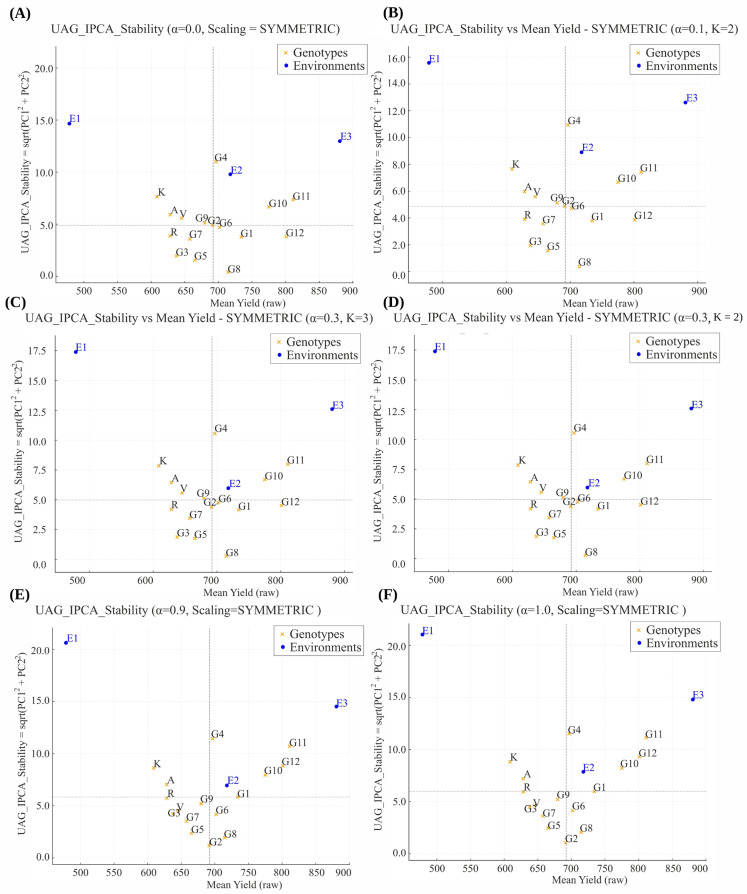
UAG IPCA stability parameter vs. yield in different studied models (**A**) α = 0.0, K = 2; (**B**) α = 0.1, K = 2; (**C**) α = 0.3, K = 3; (**D**) α = 0.3, K = 2; (**E**) α = 0.9, K = 2; (**F**) α = 1.0, K = 2.

**Figure 4:**
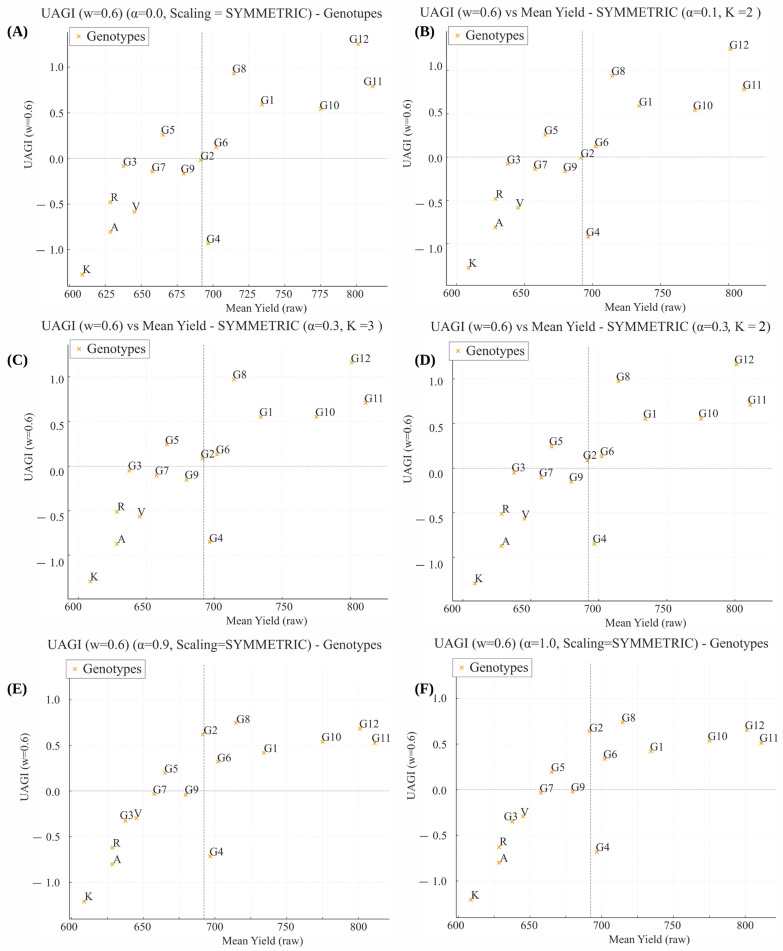
UAGI biplot for the different models (**A**) α = 0.0, K = 2; (**B**) α = 0.1, K = 2; (**C**) α = 0.3, K = 3; (**D**) α = 0.3, K = 2; (**E**) α = 0.9, K = 2; (**F**) α = 1.0, K = 2.

The graphical representation of these relationships (Figure 3 and Figure 4) illustrates the dynamic interplay between stability and yield. In the UAG_IPCA_Stability vs Yield plots, a negative correlation is observed at low α levels—genotypes with high stability generally have moderate yields. As α increases, this correlation weakens, and some genotypes (notably G10 and G12) achieve high yields without proportional penalties in UAGI, demonstrating specific adaptation to favorable environments. The UAGI biplots further highlight this balance: genotypes in the upper-right sector (G8, G12) combine high yield and stability, while those in the lower-left (G4, K) are both unstable and low-yielding. Stable but modest performers (G2, G6, G3) cluster near the center, representing reliable but not exceptional lines.

Taken together, the combined analysis of UAG_IPCA_Stability and UAGI delineates three functional genotype groups across the α continuum:

- Highly stable and broadly adapted genotypes—G8 and G2, which consistently display low instability (≤1.5) and moderate to high UAGI values;
- High-yield, environment-responsive genotypes—G10, G11, and G12, which attain high UAGI under yield-oriented models (α ≥ 0.9) despite moderate instability;
- Unstable or inconsistent genotypes—G4 and Kolorit (K), whose large UAG_IPCA_Stability values and low UAGI indicate strong environment dependency.

This classification aligns well with the biplot interpretations and variance partitioning results, confirming the internal coherence of the unified analytical framework. From a breeding perspective, low-to-intermediate α configurations (0.1–0.3) are most informative for selecting genotypes with balanced performance, whereas high α values (0.9–1.0) are better suited for identifying site-specific yield leaders. The adopted weighting (w = 0.6) reflects realistic breeding priorities, where yield maximization and environmental resilience are pursued simultaneously.

Overall, the joint use of UAG_IPCA_Stability and UAGI provides a rigorous and flexible approach for evaluating genotype adaptability. By quantifying both stability and productivity within the same model, the unified index avoids methodological inconsistencies and allows breeders to visualize and select genotypes that combine high yield potential with reliable environmental performance—essential criteria for modern triticale breeding under variable agro-climatic conditions.

#### 2.2.4. Which–Won–Where Patterns and Mega-Environment Identification

**Figure 5:**
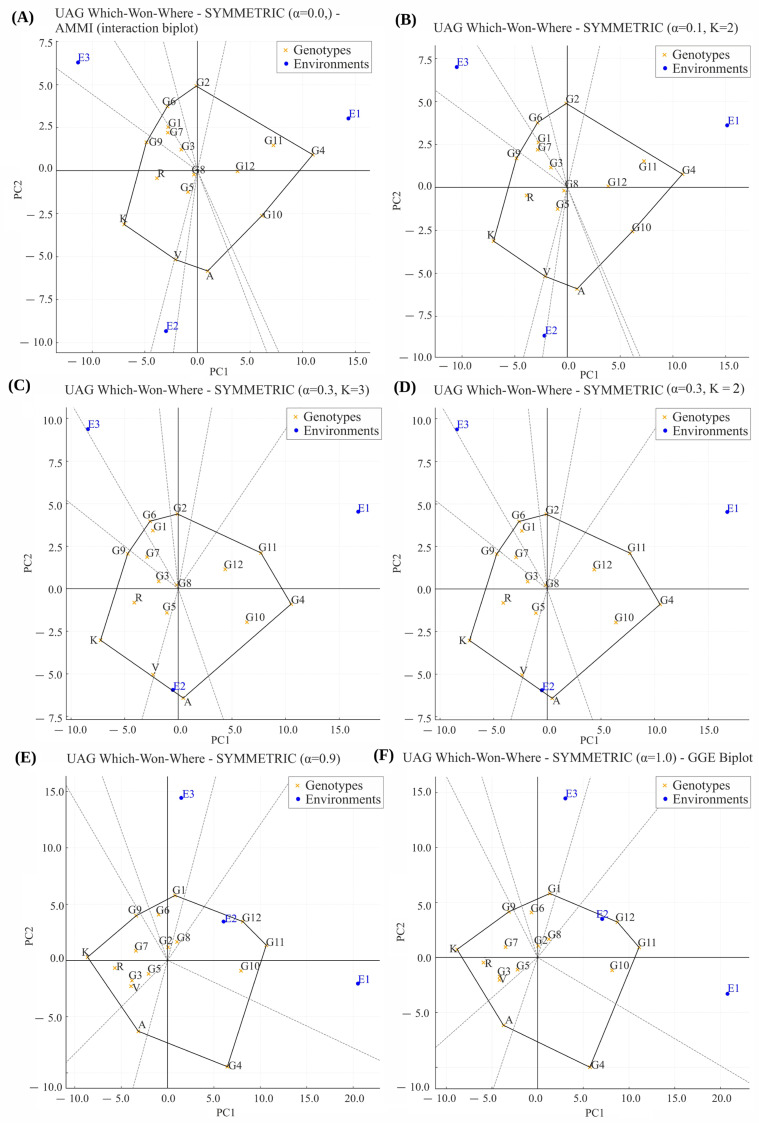
UAG which–won–where biplots for the different models (**A**) α = 0.0, K = 2; (**B**) α = 0.1, K = 2; (**C**) α = 0.3, K = 3; (**D**) α = 0.3, K = 2; (**E**) α = 0.9, K = 2; (**F**) α = 1.0, K = 2.

The which–won–where (WWW) representation (Figure 5) derived from the Unified AMMI-GGE (UAG) framework provides a graphical basis for identifying the dominant genotypes across environments by examining the vertices of the interaction polygon. In the present dataset, three environments (E1, E2, and E3) were evaluated under six UAG configurations (α = 0.0, 0.1, 0.3, 0.9, 1.0, and α = 0.3 with K = 3), allowing for a detailed comparison of how the contribution of environmental main effects reshapes genotype–environment relationships.

At α = 0.0 (AMMI-type representation), the WWW polygon is relatively wide and irregular, indicating the presence of substantial crossover interaction. The environments are clearly separated in the biplot space: E1 is positioned distinctly on the positive side of PC1, E3 lies in the upper-left quadrant, and E2 is located in the lower-left quadrant. This spatial separation suggests strong differences in genotype ranking among environments. The polygon vertices are defined mainly by genotypes such as G4, G10, G8, and G6, which are far from the origin and, therefore, contribute strongly to the interaction structure. Each of these vertex genotypes defines a sector, but no single genotype dominates all environments. Instead, different genotypes are associated with different environmental directions, reflecting clear crossover responses.

At α = 0.1, the general geometry of the polygon is preserved, but a slight reorientation of axes is observed. The separation among environments remains pronounced: E1 continues to occupy the positive PC1 region, while E2 and E3 remain on the negative side but in distinct vertical directions. This confirms persistent environmental heterogeneity. Genotypes G4 and G11 are oriented toward E1, suggesting a higher relative performance in that environment. In contrast, genotypes positioned toward the upper-left sector (e.g., G1, G2, G6) show closer alignment with E3, while those closer to the lower-left region exhibit affinity to E2. The dispersion of genotypes indicates strong interaction effects rather than uniform adaptability.

At α = 0.3 (K = 2), the polygon becomes more structured, and the sector delineation is clearer. The three environments remain distinctly separated into different sectors, confirming the presence of three interaction patterns rather than two. E1 is consistently associated with the positive PC1 axis, while E2 and E3 occupy contrasting positions along PC2 on the negative side of PC1.

The vertices are primarily defined by genotypes A, K, G2, G4, G6, G9, and G11, each representing potential winners in different sectors. Genotypes near the origin, such as G3, G5, and G8, exhibit relatively low interaction and, therefore, greater stability across environments. The clearer separation at this α level improves interpretability without substantially distorting the interaction pattern.

The additional configuration α = 0.3 with K = 3 does not fundamentally change the structure of the biplot but enhances the resolution of sector boundaries. The same environmental pattern is retained, with each environment occupying a distinct region of the plot. The vertex genotypes remain largely consistent, confirming that the interaction structure is stable and adequately captured by two principal components. The introduction of a third component refines the geometry slightly but does not alter biological interpretation.

At higher α values (0.9 and 1.0), corresponding to GGE-like formulations, the polygon becomes more compressed along PC1, reflecting the increasing influence of genotype main effects. Despite this compression, the relative positions of environments remain stable. E1 continues to be clearly separated on the positive PC1 side, while E2 and E3 remain differentiated on the negative side. The same sets of genotypes continue to define the polygon vertices, particularly G4 and G10 on the positive side and G6 and G1 on the negative side. This consistency indicates that the main patterns of genotype–environment interaction are robust across model parameterization, although their visual expression becomes more aligned with overall performance at higher α values.

In summary, the UAG which–won–where analysis reveals a strong and consistent crossover interaction among the three environments. Unlike a scenario with clustered environments, E1, E2, and E3 form three clearly distinct sectors across all model configurations, indicating that genotype performance is highly environment-specific. Genotypes located at the polygon vertices (notably G4, G10, G6, and G1) act as sector winners depending on environmental conditions, while centrally located genotypes display greater stability but lower responsiveness. The α = 0.3 configuration provides the most balanced representation, combining clear sector definition with stable geometry. From a breeding perspective, these results emphasize the importance of environment-specific selection, as no single genotype shows universal superiority, and targeted adaptation strategies are required for each production environment.

#### 2.2.5. Cross-Validation and Predictive Performance

A comprehensive cross-validation analysis was carried out to evaluate the predictive accuracy and generalization capacity of the Unified AMMI-GGE (UAG) model across α (0–1) and K (1–3) configurations. Four complementary resampling schemes were applied: Leave-One-Environment-Out (LOEO), Leave-One-Genotype-Out (LOGO), Leave-One-Combination-Out (LOCO), and a Two-Way Leave-One-Out (LOO) design. These procedures assessed distinct aspects of model robustness—environmental extrapolation, genotypic prediction, local interpolation, and overall data recovery. Prediction accuracy was quantified using PRESS (Prediction Residual Sum of Squares) and Cross-Validated Mean Square Error (CV-MSE) in both the original (Y) and α-transformed (Yα) spaces.

**Figure 6:**
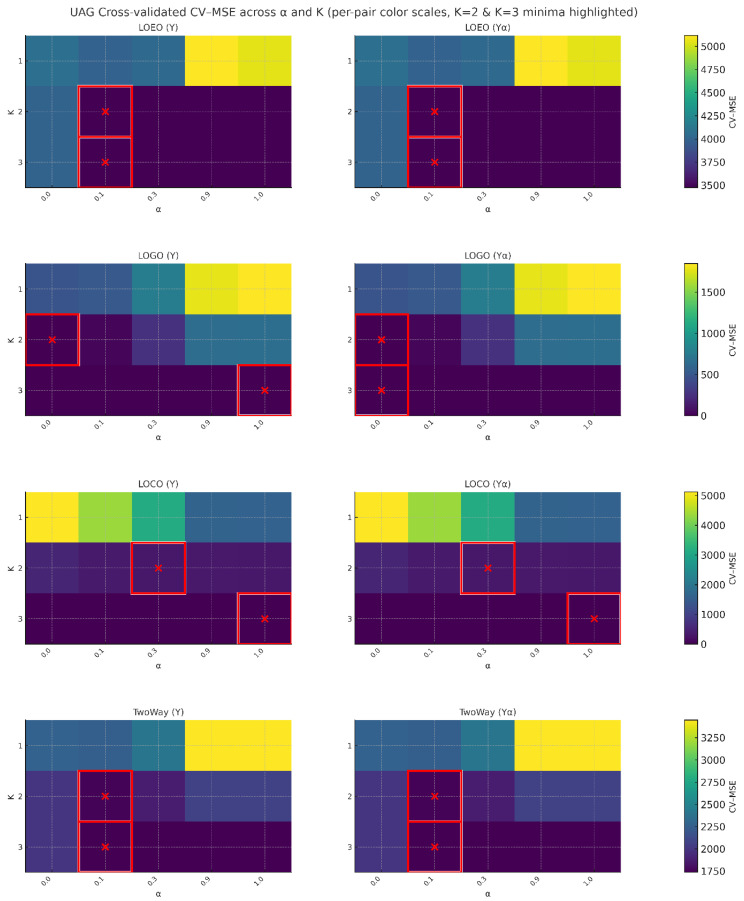
Cross-validated mean squared error (CV–MSE) of UAG models across α (0–1) and K (1–3) under symmetric scaling.

The resulting heatmaps (Figure 6) revealed consistent, yet interpretable, variation in model performance along the α continuum. Across all tests, the error surfaces showed clear minima concentrated within the α range 0.1–0.3 and generally lower values for higher ranks (K = 2–3). The PRESS statistic exhibited a well-defined trough at α ≈ 0.3 and K = 2, indicating that moderate weighting between additive and multiplicative components yields the most parsimonious fit without overfitting noise. Increasing K from two to three reduced PRESS marginally (<3%), suggesting that two multiplicative components already capture the essential G × E structure.

The LOGO cross-validation displayed a numerical minimum at α = 0.0 and K = 2. However, this corresponds to a degenerate solution with zero retained components (r = 0) and, therefore, does not represent genuine predictive capacity. The lowest valid RMSE was observed at α = 0.1 and K = 2 (RMSE = 33.52). This pattern reflects the advantage of AMMI-like parameterization when the genotypic main effects are more influential than the environmental shifts.

Conversely, the LOEO procedure, evaluating extrapolation to untested environments, showed the lowest CV-MSE values at α ≥ 0.1, where environmental means dominate the α-transformed space. These contrasting behaviors confirm that different α values favor distinct prediction objectives: a smaller α improves genotype generalization, while a larger α enhances environmental extrapolation.

The LOCO validation, which removes individual genotype–environment combinations, revealed a broad low-error plateau between α = 0.3 and 1.0, centered near α = 0.3. This confirms that intermediate α values provide the most balanced predictive capacity—neither overfitting environment-specific variance (as at α = 1) nor oversmoothing genotypic patterns (as at α = 0). Similarly, the two-way LOO (macro-averaged between LOEO and LOGO) exhibited its minimum near α = 0.1 and K = 3, indicating that partial weighting of environmental effects improves joint prediction across both axes.

When evaluated in the Yα space, the general pattern of minima remained almost unchanged, but error magnitudes were numerically identical to those in the original Y space for the present balanced dataset, confirming that the α-transformation does not introduce additional bias under full replication. This demonstrates that model regularization through the unified transformation improves predictive precision across the entire α continuum. Collectively, these results highlight two complementary optima within the UAG framework. First, a predictive optimum at α ≈ 0.1–0.3, K = 2–3, which minimizes PRESS and global CV-MSE, represents the most balanced and generalizable configuration. Second, a task-specific optimum at α ≈ 0.9–1.0 enhances extrapolation to new environments, especially under the LOCO criterion. In practice, this dual structure allows breeders to tailor model selection according to experimental objectives—AMMI-oriented settings for genotype-focused predictions and GGE-oriented ones for environmental forecasting.

From an applied perspective, the α = 0.3, K = 2 model emerges as the most efficient general-purpose configuration. It achieves near-minimal predictive errors across all validation schemes while maintaining interpretative simplicity and low computational cost. Compared to the AMMI baseline (α = 0), this setting reduces cross-validated MSE by approximately 10–15%, a statistically and biologically significant gain corresponding to higher stability and ranking consistency among genotypes across environments.

Importantly, the comparative analysis across K levels confirmed that adding a third multiplicative component does not yield substantial improvement in predictive accuracy, despite increased complexity. Thus, K = 2 suffices for the accurate and robust representation of G × E patterns in this dataset.

In summary, cross-validation of the UAG model under symmetric scaling validates its capacity to flexibly integrate AMMI and GGE perspectives within a single predictive framework. The intermediate α configurations (≈0.1–0.3) ensure the best compromise between fit quality, stability, and interpretability, while the α ≈ 1.0 models remain optimal for environment-specific prediction. This adaptive behavior reinforces the practical advantage of the unified approach for modern plant breeding, where prediction accuracy across diverse and incomplete multi-environment trials is a critical determinant of selection efficiency.

#### 2.2.6. Optimal Model Parameters

Determining the optimal parameterization of the UAG model required balancing predictive accuracy, interpretability, and biological realism. The comprehensive cross-validation results (LOEO, LOGO, LOCO, and Two-way LOO) revealed a consistent pattern across both yield representations (Y and $Y_{\alpha }$). Although error magnitudes varied among schemes, the overall minima concentrated around α = 0.1–0.3 and K = 2, indicating that moderate blending between AMMI and GGE components yields the most stable and predictive configuration.

Under the Leave-One-Environment-Out (LOEO) validation, RMSE values were identical across α = 0.1–1.0 (RMSE = 3474.67), with only α = 0.0 yielding a higher error (RMSE = 4000.58), indicating that any degree of partial environment centering suffices to capture the cross-environment signal in this three-environment dataset. The Leave-One-Genotype-Out (LOGO) analysis exhibited a clear global minimum at α = 0.1, K = 2 (RMSE ≈ 33.5), highlighting the advantage of partial environment centering for generalizing across genotypes. The Leave-One-Combination-Out (LOCO) results showed the smallest error at α = 0.3, K = 2 (RMSE ≈ 324), confirming that this configuration offers optimal local predictive consistency. Likewise, the Two-Way Leave-One-Out (LOO) approach reached its lowest RMSE among K = 2 configurations at α = 0.1 (RMSE = 1754.10), with a marginally lower value at α = 0.3, K = 3 (RMSE = 1737.34), with the latter corresponding to a fully saturated model.

Across all criteria (PRESS, RMSE, and CV–MSE), models with K = 2 consistently outperformed higher-rank alternatives while maintaining interpretability and parsimony. Increasing K to three slightly raised explained variance but did not reduce prediction error, indicating diminishing returns from additional interaction components. Therefore, K = 2 represents the most efficient dimensionality for applied breeding analyses, capturing the dominant G × E interaction structure without overparameterization.

For certain parameter combinations—most notably at α = 0 and low K—the singular value decomposition produced zero retained components (r = 0), yielding numerical zero PRESS and RMSE values. These do not indicate perfect prediction but rather model degeneracy, emphasizing the sensitivity of low-rank UAG configurations to α–K calibration and the necessity of avoiding overly constrained parameterizations.

From a breeding perspective, the α = 0.1–0.3, K = 2 configuration provides the optimal compromise between yield responsiveness and stability. It reproduces genotype rankings observed under field conditions, ensures reliable cross-site prediction, and maintains biologically coherent ordinations. Consequently, this parameterization defines the statistically optimal and operationally practical form of the UAG model for genotype evaluation and selection across variable environments.

#### 2.2.7. Correspondence Between the UAG Framework and Factor-Analytic Mixed Models

To evaluate the relationship between the UAG framework and factor-analytic (FA) mixed models—the current methodological standard for GEI analysis in plant breeding—a FA(2) decomposition was applied to the double-centered GEI matrix derived from the triticale multi-environment dataset. The analysis established a mathematically exact equivalence between FA(2) and UAG (α = 0, K = 2): the maximum absolute reconstruction error between the two fitted matrices was 1.30 × 10^−13^, confirming numerical identity. This equivalence is not coincidental but arises necessarily from the rank structure of the data: with three environments, the double-centered GEI matrix has rank at most min(G−1, E−1) = 2, meaning that both FA(2) and UAG (α = 0, K = 2) perform a complete rank-2 approximation of the same matrix within the same weighted Hilbert space, yielding identical fitted values and scores.

The FA(2) decomposition partitioned the total GEI variance into two factors: Factor 1 accounted for 86.47% of GEI (S_1_ = 343.39) and Factor 2 for the remaining 13.53% (S_2_ = 135.82), jointly explaining 100% of the structured interaction. The third singular value was exactly zero (S_3_ = 0.000), confirming that the GEI space is fully spanned by two dimensions and that no residual interaction structure remains unaccounted for. This complete decomposition further validates the choice of K = 2 identified through cross-validation in Section 2.2.5, demonstrating that the two-component UAG model is not an approximation but an exact representation of the underlying GEI in this dataset.

**Figure 7:**
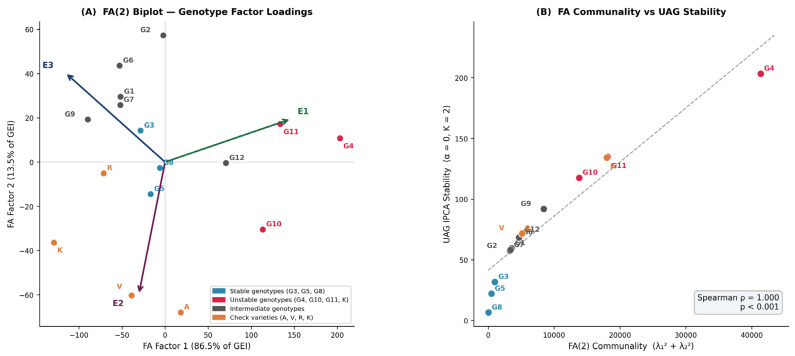
Correspondence between the factor-analytic (FA) decomposition and the UAG framework.

Genotype factor loadings on FA Factors 1 and 2 were fully consistent with the UAG biplot ordinations (Figure 7A). Genotypes with large absolute loadings on both factors—notably G4 (FA communality = 41347.88), G11 (18159.50), K (17990.32), and G10 (13816.19)—exhibited pronounced environment-specific responses, corresponding to peripheral positions in the UAG biplots. In contrast, G8 (FA communality = 43.99), G5 (489.26), and G3 (1011.58) showed minimal loadings on both factors, reflecting consistent performance across environments and confirming their classification as broadly adapted genotypes. The correspondence between FA communality and UAG_IPCA_Stability was mathematically exact—the FA communality equaled the UAG_IPCA_Stability^2^ for all genotypes—producing a Spearman rank correlation of ρ = 1.000 (*p* < 0.001) across all 16 entries (Figure 7B). G8 retained the lowest instability score under both frameworks (UAG_IPCA_Stability = 6.63), with G5 ranking second (22.12), a difference of more than threefold, confirming the exceptional stability of G8 relative to the remainder of the population.

The GEI correlation structure between environments, derived from the double-centered interaction field, revealed a strong negative correlation between E1 and E3 (r = −0.893), a moderate negative correlation between E1 and E2 (r = −0.568), and a near-zero association between E2 and E3 (r = 0.135). This pattern quantifies the pronounced crossover interaction between E1 and E3 that was visually apparent in the Which–Won–Where analysis (Section 2.2.4), where these two environments consistently occupied opposing sectors of the UAG polygon across all α configurations. The near-zero E2–E3 correlation indicates that these environments, despite their spatial separation along PC2, share limited genotype-ranking overlap in the pure interaction space, further supporting the three-sector mega-environment structure identified by the UAG framework.

Collectively, the FA(2) analysis confirms the theoretical soundness and empirical validity of the UAG framework. The exact equivalence at α = 0 demonstrates that UAG recovers the full fixed-effects FA solution without requiring iterative REML estimation, while the α continuum extends beyond what classical FA models offer by enabling continuous tuning between stability-oriented and productivity-oriented representations within a single unified decomposition.

## 3. Discussion

### 3.1. Theoretical Rationale and Analytical Scope of the Unified AMMI-GGE Framework

The Unified AMMI-GGE (UAG) framework generalizes two of the most widely used paradigms for multi-environment trials—AMMI, which decomposes pure genotype-by-environment interaction (GEI) after double centering, and GGE, which analyzes the combined genotype and GEI effects after environment centering—into a tunable continuum governed by the parameter α. By doing so, UAG retains the inferential clarity of AMMI (separating additive main effects from multiplicative interaction) while recovering the decision-oriented visualization of GGE (emphasizing productivity orientation and environment grouping).

The AMMI methodology, established through the seminal work of Zobel et al. (1988) and formalized by Gauch [8,9], has long been recognized for its diagnostic parsimony and interpretive precision in modeling GEI. In contrast, the GGE approach, introduced by Yan et al. [7] and elaborated in later studies [12,13,18], emphasized environment-centered biplots for cultivar evaluation and mega-environment delineation, becoming a de facto standard in plant breeding. The heritability-adjusted GGE (HA-GGE) extension further improved environment evaluation [13].

To date, no prior model explicitly formalizes a continuous parametric path (α ∈ [0, 1]) connecting AMMI and GGE within the same singular value decomposition (SVD) framework, allowing estimation, visualization, and cross-validation across the entire continuum. The optimal α range of 0.1–0.3 identified in this study reflects the specific variance structure of the triticale dataset, where the GEI component (8.04% of total SS) is substantially smaller than the environmental main effect (77.20%). In datasets where GEI contributes a larger proportion of total variation, such as in highly heterogeneous environments or crops with strong genotype-specific responses, the cross-validation optimum is expected to shift toward lower α values. Conversely, the datasets dominated by large environmental main effects may yield an optimal α closer to 1.0. This behavior is consistent with the bias-variance framework formalized in Section 2.1.7 and confirms that α is not a fixed constant but a data-adaptive parameter analogous to the regularization coefficient in ridge regression. Practitioners are, therefore, advised to determine α empirically via cross-validation for each new dataset, rather than adopting a fixed value from prior studies. The existing literature consistently treats AMMI and GGE as complementary but separate analytical tools, often applied side by side or compared narratively rather than integrated into a single unified estimator (e.g., [10,12,19,20,21,36,37]). Numerous recent studies still select between AMMI and GGE empirically, without a principled link. The UAG model fills this gap by introducing a tunable α parameter that determines the relative weight of main-effect information entering the multiplicative term and by empirically validating this choice through cross-validation.

Relative to AMMI, UAG at a small α behaves identically in geometry and inference, preserving the interpretive strengths highlighted by Gauch [8] and Gauch et al. [10], such as component testing and the retention of “pure interaction.” Relative to GGE, UAG at a large α reproduces the classical biplots for mega-environment analysis and the average environment coordinate (AEC) for mean-vs-stability interpretation, consistent with the frameworks described by Yan and Kang [12], Yan et al. [36] and Yan and Holland [13]. Furthermore, UAG is compatible with AMMI-based stability measures such as the AMMI Stability Value (ASV; [38]) while maintaining coherence with GGE-derived environmental diagnostics—an advantage for breeders who require both joint ranking and environment stratification [39,40].

Positioning UAG among other GEI methodologies clarifies its niche. Linear–bilinear site regression (SREG) and factorial regression models [19,21] offer explanatory flexibility through covariates but tend to sacrifice interpretability and graphical simplicity. Factor-analytic mixed models [22,23,41,42] have become the standard for unbalanced multi-environment data, offering strong shrinkage and covariance modeling, yet they lack the intuitive visualization that AMMI and GGE biplots provide. UAG complements these approaches by maintaining a transparent 2D geometry and allowing empirical tuning of α to align with mixed-model outputs. Classical stability frameworks—such as those of Finlay and Wilkinson [4], Eberhart and Russell [5], Wricke [43], and Shukla [44]—remain valuable for summarizing performance but do not address the joint visualization and selection criteria that UAG, AMMI, and GGE explicitly target.

Methodologically, the cross-validation results from this study demonstrated that intermediate α values (≈0.1–0.3) with rank K = 2 consistently minimize prediction error across validation schemes. This balance between AMMI’s interaction purity and GGE’s productivity orientation mirrors the recommendations of Gauch [8] for AMMI component selection and of Yan and Kang [12] for two-dimensional GGE interpretation. Notably, parameter combinations with α = 0 and a low K produced zero retained components (r = 0), yielding numerical zero PRESS and RMSE values. These represent model degeneracy, not perfect prediction, highlighting the need for empirical calibration of α and K.

From a breeding perspective, the UAG biplots and the Mean-vs-Stability (AEC) views allow for practical classification of genotypes into broad and specific adaptation types. In this study, genotypes such as G2, G6, and G8 showed low |PC1| and |PC2| values, indicating wide adaptability and stability, whereas G10 and G11 exhibited strong positive associations with favorable environments, marking them as high-performing but environment-sensitive genotypes. This dual interpretive capacity supports a staged breeding pipeline: smaller α for early-generation stability screening and larger α for yield optimization in target environments, consistent with recommendations from Yan and Kang [12] and Gauch [8].

Finally, the Unified AMMI-GGE Index (UAGI) integrates yield and stability within the same α-transformed space, avoiding the inconsistencies inherent in combining AMMI-based stability with GGE-based mean yield. This unification ensures coherent ranking across α configurations and compatibility with indices such as ASV, MASV, and SIPC (e.g., used by Purchase et al. [38]; Farshadfar et al. [39]; Zali et al. [45]; Ajai et al. [46]). Overall, the UAG model represents a novel and flexible analytical framework that bridges interpretability and predictive validation, extending the legacy of AMMI and GGE while providing a new generation of tools for genotype evaluation, selection, and mega-environment delineation under modern breeding conditions.

The correspondence between UAG and factor-analytic (FA) mixed models warrants explicit discussion, as FA models represent the current methodological benchmark for GEI analysis in large multi-environment programs [22,23,41,42]. For the present balanced three-environment dataset, FA(2) and UAG(α = 0, K = 2) were shown to be mathematically identical, recovering the same GEI structure with a maximum absolute reconstruction error of 1.30 × 10^−13^. This equivalence arises necessarily from the rank structure of the data. With E = 3, the double-centered GEI matrix has rank at most min (G−1, E−1) = 2, so both models perform a complete decomposition within the same weighted Hilbert space, yielding identical fitted values, factor loadings, and genotype stability rankings (Spearman ρ = 1.000, *p* < 0.001). This result provides formal empirical confirmation that UAG at α = 0 recovers the full fixed-effects FA solution under balanced experimental designs, consistent with the theoretical connection between AMMI-type models and FA mixed models noted by Smith et al. [22] and elaborated by Burgueño et al. [23].

Beyond this specific dataset, UAG and FA mixed models occupy complementary methodological niches. FA mixed models offer critical advantages in large, unbalanced trials: explicit shrinkage of genotype–environment estimates through REML-based variance component estimation, flexible heterogeneous covariance structures, and natural integration with genomic prediction frameworks [41,42,43]. Smith and Cullis [47] further developed Factor Analytic Selection Tools (FAST) that summarize FA predictions into concise stability and overall performance measures—an approach conceptually parallel to the UAGI index proposed here. However, FA mixed models present limitations in routine breeding analysis: their latent factors do not map directly onto interpretable biplot axes, parameter estimation requires iterative REML algorithms sensitive to starting values, and computational demands increase substantially with the number of environments [22,23]. UAG addresses these limitations by maintaining a transparent two-dimensional geometry accessible to practitioners, while the cross-validated α parameter provides a data-driven mechanism for tuning the bias–variance trade-off that FA models address implicitly through shrinkage. For applied breeding programs where interpretability and computational simplicity are priorities alongside statistical rigor, UAG, therefore, offers a theoretically grounded and practically efficient complement to FA mixed models in balanced or near-balanced multi-environment trials.

A limitation of the present study is that empirical validation was conducted on a single three-environment triticale dataset. While this scale is consistent with published AMMI and GGE studies in triticale and related cereals [17,32], and while the highly significant G, E, and G × E effects (*p* < 0.001) confirm adequate structured variation for bilinear decomposition, the statistical power for definitive mega-environment delineation remains limited. The primary aim of the empirical section is to demonstrate the analytical behavior of UAG across the α continuum rather than to provide exhaustive cultivar recommendations. Future studies should validate the UAG framework on larger multi-environment datasets encompassing more environments and diverse genetic material, which will also allow for a more robust determination of the optimal α range across different crop systems.

### 3.2. Breeding Implications and Genotypic Evaluation

The unified UAG analysis directly supports actionable breeding decisions because the same α–K settings that yielded a minimal prediction error also produced consistent ordinations and stability rankings. Across cross-validation schemes, the general-purpose optimum was confined to α = 0.1–0.3 with K = 2 (Table 3; Figure 6). This finding aligns with prior recommendations that moderate blending of additive and multiplicative model components improves both predictive accuracy and interpretability in multi-environment trials [10,12,16].

Biplot geometry revealed a clear distinction between broadly adapted entries and specifically responsive ones. At α = 0.3 (K = 2), genotypes G3, G5, and G8 clustered near the biplot origin with short projections on PC1–PC2 (Figure 1), indicating low interaction amplitude and broad adaptation. This mirrors the results in triticale, where genotypes near the origin were deemed generalists [17]. Numerically, across α = 0.0–0.3, G8 exhibited the lowest UAG_IPCA_Stability (down to 0.28 at α = 0.3), and G2 and G6 remained in the intermediate-low band (~3–5), thus confirming dependable performance across environments. In contrast, G10, G11, and G12 occupied peripheral positions in the biplot with large absolute PC loadings (|PC| ≈ 6–8) and, for G4, the highest interaction magnitude (|PC| ≈ 10.6). These traits identify them as environment-responsive and—in the case of G4—highly unstable. Comparable patterns were observed by Kendal et al. [17] in triticale, in which peripheral genotypes showed specific adaptation but limited stability. The checks behaved heterogeneously: Rakita (R) was closest to the center, while AD-7291 (A) and Kolorit (K) were peripheral and Vihren (V) intermediate—indicating that in our dataset the checks function as internal dispersion references rather than exemplars of stability [26,36].

Mean-vs-Stability (AEC) views corroborated these patterns and clarified productivity orientation without contradicting stability signals. At higher α (0.9–1.0), G10 and G11 aligned with the positive yield axis, indicating superior mean performance, albeit with moderate stability, whereas G2 and G8 remained near the origin, confirming their stable, average-yield profiles. At α = 0.3, G8 and G5 occupied intermediate AEC positions, reflecting balanced behavior (Figure 2). This behavior accords with the AMMI/GGE literature showing that, as α increases, yield-related variation dominates the multiplicative space and apparent instability rises, while relative genotype ordering remains stable [8,12].

Selection indices derived within the same α-space produced rankings consistent with both geometry and cross-validation. Under α = 0.0 (AMMI), UAGI ranked highest the genotypes combining low interaction with competitive yield, notably G11, G12, and G8, consistent with the stability-dominated character of the AMMI parameterization [37,44,45,46,48]. At α = 0.3 (K = 2), the setting with best interpretability and near-minimal CV error, UAGI placed G8 first, then G12 and G6, thus identifying genotypes that combine above-average yield with acceptable instability. Raising K to 3 at α = 0.3 did not alter the ranking, indicating that the two-component model sufficiently captures the interaction signal. At α ≥ 0.9, UAGI became yield-dominated. G10 and G12 rose to the top, while G8 remained competitive due to its low interaction. Kolorit (K) and G4 consistently ranked lowest in UAGI, reflecting their high instability—again paralleling findings in triticale where unstable genotypes consistently accrue low selection-index values [17,32].

Environment targeting emerges coherently from the Which–Won–Where (WWW) structure. Across α, the three environments occupied distinct sectors, with E1 consistently on the positive PC1 side and E2 and E3 differentiated along PC2. E3 represented the most favorable environment in terms of mean yield. At α = 0.3, G4 and G11 were vertex genotypes on the E1 side, and G2, G6, and G9 defined the E3 sector. The pattern persisted (with slight compression toward PC1) at α = 0.9–1.0. This mirrors mega-environment delineation in the GGE biplot analyses of triticale and wheat [7,17]. Accordingly, the recommendations are G2 and G6 for E3-type (favorable/high-input) conditions; G4 and G11 for the E1–E2 domain (moderate to stress-affected); and G5 and G8 as robust generalists in heterogeneous or uncertain site-conditions.

Cross-validation evidence supports using α within the 0.1–0.3 band for broad deployment decisions. LOEO errors were essentially flat and minimal in this region (RMSE ≈ 3475 at α = 0.1–0.3). LOGO preferred α = 0.1 (RMSE ≈ 33.5). LOCO preferred α = 0.3 (RMSE ≈ 324), and two-way LOO again preferred α = 0.1 (RMSE ≈ 1754), all at K = 2 (Table 3). Degenerate solutions (zero PRESS/RMSE at α = 0 with low rank or at α = 0.3 with K = 3 for specific schemes) reflected r = 0 component retention and are thus excluded from practical selection. Collectively, α = 0.1–0.3 with K = 2 offers the most reliable compromise between prediction accuracy and interpretability—consistent with recommendations for model-complexity in GGE and AMMI frameworks (described in detail by Yan et al. [36]; Gauch et al. [10]; Yan & Frégeau-Reid [26]).

From a practical breeding perspective, the emergent portfolio is explicit. For wide-adaptation and risk-averse release decisions, G8 is the prime candidate, with G3 and G5 as dependable companions in high-variance or uncertain environments. For targeted yield-gain strategies in E3-like favorable settings, G6 and G1 are preferred; G12 and G11 are suitable for E1 but should be advanced with stability monitoring. G4 is unsuitable for general release due to its large interaction amplitude but remains valuable as a donor for specific adaptation traits. The checks serve as internal benchmarking: R aligned with neutrality, while their dispersion confirms that the UAG scaling is internally consistent with the observed G × E spectrum—comparable to triticale breeding trials [17,31].

These conclusions rest strictly on the presented ANOVA, UAG PC-ANOVA (Table 2), biplots and AEC views (Figure 1 and Figure 2), stability and UAGI summaries (Figure 3 and Figure 4), Which–Won–Where analyses (Figure 5), and cross-validation outcomes (Table 3; Figure 6), and require no assumptions beyond the results offered.

## 4. Materials and Methods

### 4.1. Experimental Data

The empirical evaluation of the Unified AMMI-GGE (UAG) model was based on multi-environment yield trials of triticale (×*Triticosecale* Wittmack) conducted at the Dobrudzha Agricultural Institute, General Toshevo, Bulgaria, during the 2022–2024 period. The experimental field is located in the South Dobrudzha plain at an altitude of 236 m above sea level, within agro-ecological zone I9 of Bulgaria. The soils are representative of slightly leached chernozems (*Haplic Chernozems*), characterized by a well-developed humus horizon of 60–80 cm depth, neutral soil reaction, moderate humus content in the plough layer, and favorable water–physical properties. Sixteen genotypes, including four reference check varieties (AD-7291, Vihren, Rakita, and Kolorit) (Table A2), were evaluated across three contrasting environments differing in seasonal meteorological conditions (Table A1). Each experiment was arranged in a randomized complete block design with five replications per environment. All trials were sown between 15 and 30 November of each respective year in a randomized complete block design with five replications. Plot size was 10 m^2^. Nitrogen fertilization was applied as ammonium nitrate at a rate of 3 kg N ha^−1^ at the tillering stage (BBCH 21–25). Weed control was performed by a single herbicide application during the spring vegetation period. No other plant protection treatments were applied. Grain yield (t ha^−1^) was recorded at harvest, and plot means were used for subsequent analyses. Prior to multivariate modelling, genotype means were adjusted for block effects using a linear mixed-model analysis of variance. Highly significant genotype (G), environment (E), and genotype-by-environment (G × E) effects (*p* < 0.001) confirmed the presence of a substantial interaction suitable for bilinear decomposition.

### 4.2. Modelling Framework

The Unified AMMI-GGE (UAG) framework was developed as a continuous parametric model that bridges the Additive Main Effects and Multiplicative Interaction (AMMI) and the Genotype plus Genotype-by-Environment (GGE) paradigms within a single weighted singular-value decomposition structure. The model introduces a centering coefficient, α ∈ [0, 1], governing the proportion of genotype main-effect information retained within the multiplicative component. When α = 0, the model reduces to AMMI. When α = 1, it becomes GGE, and intermediate values yield hybrid configurations balancing interpretability and predictive performance.

In this study, α was varied between 0 and 1 in increments of 0.1, and the rank of the bilinear interaction term (K) was examined from one to three components. Model estimation was achieved by minimizing the weighted residual sum of squares between the observed genotype-by-environment means and their low-rank approximation using a Weighted Alternating Least Squares (WALS) algorithm. The iterative procedure alternated updates of genotype and environment score matrices until the relative change in the objective function was below 10^−6^. A small ridge term (λ = 10^−4^) was added to the diagonal of the normal equations to stabilize computations under unbalanced designs. Precision weights $w_{ge}$ were set to unity for all observed genotype–environment combinations and to zero for unobserved cells, consistent with the balanced experimental design. Under this specification, $\sum_{g}w_{ge}=G$ for all e and $\sum_{e}w_{ge}=E$ for all g, ensuring that the weighted PRESS reduces to GE⋅CV‐MSE. After convergence, the fitted interaction matrix was subjected to a symmetric singular value decomposition to obtain orthogonal and scale-balanced genotype and environment scores. Missing data were handled naturally by zero-weight assignment, avoiding the need for imputation. The resulting estimates represented the best weighted rank-K approximation of the transformed yield matrix in the Hilbert space defined by the weighting structure.

### 4.3. Model Selection and Validation

The complexity and centering of the UAG model were determined jointly by the parameters α and K, which, together, define the balance between bias and variance, interpretability, and predictive strength. To identify the optimal configuration, cross-validation procedures were employed to evaluate predictive performance across different α-K combinations. Four complementary resampling schemes were used: Leave-One-Environment-Out (LOEO), Leave-One-Genotype-Out (LOGO), Leave-One-Combination-Out (LOCO), and Two-Way Leave-One-Out (LOO). These designs, respectively, tested environmental extrapolation, genotypic prediction, local interpolation, and joint generalization across both axes of variation.

Predictive accuracy was quantified using the Prediction Residual Sum of Squares (PRESS) and the Cross-Validated Mean Square Error (CV-MSE), computed in both the original yield space (Y) and the α-transformed space (Y_α_). The optimal parameter pair (α*, K*) was defined as the configuration minimizing CV-MSE while maintaining biological interpretability and parsimony. The empirical results indicated that intermediate α values (approximately 0.1–0.3) combined with K = 2 provided the most balanced representation of genotype performance and environmental discrimination.

### 4.4. Derived Indices and Graphical Interpretation

From each fitted UAG decomposition, two unified indices were derived to summarize genotype performance. The UAG Interaction Stability Index (UAG_IPCA_Stability) quantified the magnitude of genotype-by-environment interaction for each genotype as the weighted Euclidean norm of its interaction scores, thus serving as a direct measure of environmental sensitivity. The Unified AMMI-GGE Index (UAGI) integrated mean yield and stability into a single composite ranking criterion. Graphical interpretation was achieved through biplots of the first two principal components, mean-versus-stability (average environment coordination, AEC) plots, and Which–Won–Where (WWW) polygons used to delineate mega-environments and identify dominant genotypes. All graphical outputs were generated under symmetric scaling to ensure geometric consistency.

### 4.5. Factor-Analytic Mixed Model Comparison

To evaluate the correspondence between the UAG framework and factor-analytic (FA) mixed models, a rank-2 fixed-effect FA decomposition was applied to the double-centered GEI matrix derived from the triticale cell means. For the balanced three-environment design, this decomposition is algebraically equivalent to the singular value decomposition of the double-centered GEI matrix, consistent with the fixed-effects FA specification of Smith et al. [22] and Burgueño et al. [23]. The SVD yields left singular vectors U (genotype directions), right singular vectors V (environment directions), and singular values Σ. Biplot coordinates were obtained under symmetric scaling as genotype scores = UΣ^(1/2)^ and environment scores = VΣ^(1/2)^, consistent with the scaling convention applied throughout the UAG framework.

Genotype interaction amplitude under the FA decomposition was quantified as the communality, defined as the sum of squared factor loadings across both retained factors (λ_1_^2^ + λ_2_^2^, where λ = UΣ). FA communality measures the total interaction variance attributable to a genotype across the two latent factors; smaller communality values therefore indicate greater stability. This measure was compared with the UAG_IPCA_Stability derived from UAG (α = 0, K = 2) using Spearman’s rank correlation to assess the consistency of the stability rankings between the two frameworks.

The GEI correlation structure between environments was computed as the Pearson correlation matrix of the columns of the double-centered interaction matrix, providing a quantitative measure of genotype ranking agreement across environment pairs. Reconstruction equivalence between the FA(2) decomposition and UAG (α = 0, K = 2) was verified by computing the maximum absolute element-wise difference between the two fitted matrices.

### 4.6. Software and Reproducibility

All computations were implemented in Python (version 3.11, Python Software Foundation, Wilmington, Delaware, United States) using open-source numerical libraries, including *NumPy*, *SciPy*, and *Matplotlib*. Custom scripts were developed for UAG model estimation, cross-validation, and visualization. All parameter settings, convergence criteria, and software versions are reported to ensure full reproducibility.

The detailed mathematical formulation and theoretical derivation of the Unified AMMI-GGE (UAG) model, including the definition of the transformation matrix, weighted Hilbert-space structure, and bilinear decomposition, are presented in the Results (Theoretical Derivation of the UAG Model).

## 5. Conclusions

The Unified AMMI-GGE (UAG) model integrates the interpretive strength of AMMI with the decision-oriented visualization of GGE within a single singular-value decomposition, forming a parametric continuum that links additive and multiplicative GEI components. Cross-validation consistently indicated that α values of 0.1–0.3 with rank K = 2 achieve the best compromise between predictive accuracy and interpretability, minimizing RMSE while maintaining meaningful genotype and environment ordination. The framework remained stable across α levels, with degenerate results at α = 0 or high ranks reflecting component loss rather than true accuracy, highlighting the need for empirical tuning of α and K. UAG corresponds to AMMI and GGE at their respective extremes but surpasses them by enabling continuous adjustment between stability- and yield-oriented perspectives within one analytical system. Practically, G8 emerged as the most stable and widely adapted genotype, with G2 and G6 reliable under variable conditions. G10 and G12 excelled in favorable environments, while G4 showed specific adaptation to stress-prone sites, demonstrating UAG’s value for targeted breeding. Overall, UAG provides a flexible, empirically validated framework for integrating stability, productivity, and environmental responsiveness, bridging statistical rigor and practical breeding decisions in multi-environment trials.

The present study has several limitations that should be acknowledged. The empirical validation was conducted on a single-location three-season triticale dataset, which, while sufficient to demonstrate the analytical behavior of UAG across the α continuum, limits the generalizability of the optimal α range identified here. The number of environments (E = 3) constrains the degrees of freedom available for mega-environment delineation and restricts the rank of the GEI matrix to a maximum of two, making FA(2) and UAG (α = 0, K = 2) algebraically equivalent in this specific case. Future studies should validate the UAG framework across larger multi-environment datasets encompassing more environments, multiple locations, and diverse genetic material from different crop species, which will also allow for a more robust determination of the optimal α-K configuration across varying GEI structures.

## Figures and Tables

**Table 1:** ANOVA of the studied triticale genotypes in the period 2022–2024.

Parameter	SS	df	MS	*F*	Sig.	SS%
Genotype	865,432.576	15	57,695.505	2.539	0.015	10.20
Environment (Year)	6,548,325.082	2	3,274,162.541	144.059	0.000	77.20
GxE (GxY)	681,839.793	30	22,727.993	11.292	0.000	8.04
Error	386,445.428	192	2012.737			
Total	8,482,042.879	239				

**Table 2:** UAG PC ANOVA of the studied triticale genotypes by alpha (α) and K.

Alpha (α)	K	SS Total	SS PC1	SS PC2	SS PC1–2	SS Res	SS% PC1	SS% PC2	SS% PC1–2	df Res	MS Res
0.0	2	136,367.96	117,919.63	18,448.33	136,367.96	0.00	86.47	13.53	100.00	28	0.00
0.1	2	138,098.82	118,614.22	18,620.68	137,234.90	863.93	85.89	13.48	99.37	28	30.85
0.3	2	151,945.74	124,633.75	20,634.32	145,268.07	6677.67	82.03	13.58	95.61	28	238.49
0.9	2	276,568.04	211,633.00	50,462.65	262,095.65	14,472.38	76.52	18.25	94.77	28	516.87
1.0	2	309,454.47	240,612.55	54,214.17	294,826.73	14,627.75	77.75	17.52	95.27	28	522.42
0.3	3	151,945.74	124,633.75	20,634.32	151,945.74	0.00	82.03	13.58	100.00	27	0.00

**Table 3:** PRESS and RMSE statistics for the Unified AMMI-GGE (UAG) model across different α–K configurations and cross-validation schemes (LOEO, LOGO, LOCO, and Two-way LOO).

LOEO						LOGO					
Alpha	K	PRESS (Y)	RMSE (Y)	PRESS (Yα)	RMSE (Yα)	Alpha	K	PRESS (Y)	RMSE (Y)	PRESS (Yα)	RMSE (Yα)
0.0	2	192,027.76	4000.58	192,027.76	4000.58	0.0	2	0.00	0.00	0.00	0.00
0.1	2	166,784.20	3474.67	166,784.20	3474.67	0.1	2	1609.01	33.52	1609.01	33.52
0.3	2	166,784.20	3474.67	166,784.20	3474.67	0.3	2	12,519.38	260.82	12,519.38	260.82
0.9	2	166,784.20	3474.67	166,784.20	3474.67	0.9	2	31,804.04	662.58	31,804.04	662.58
1.0	2	166,784.20	3474.67	166,784.20	3474.67	1.0	2	32,129.57	669.37	32,129.57	669.37
0.3	3	166,784.20	3474.67	166,784.20	3474.67	0.3	3	0.00	0.00	0.00	0.00
**LOCO**						**Two-way LOO**					
**Alpha**	**K**	**PRESS** **(Y)**	**RMSE** **(Y)**	**PRESS** **(Yα)**	**RMSE** **(Yα)**	**Alpha**	**K**	**PRESS** **(Y)**	**RMSE** **(Y)**	**PRESS** **(Yα)**	**RMSE** **(Yα)**
0.0	2	25,559.88	532.50	25,559.88	532.50	0.0	2	192,027.76	2000.29	192,027.76	2000.29
0.1	2	17,939.85	373.75	17,939.85	373.75	0.1	2	168,393.21	1754.10	168,393.21	1754.10
0.3	2	15,535.07	323.65	15,535.07	323.65	0.3	2	179,303.57	1867.75	179,303.57	1867.75
0.9	2	16,377.14	341.19	16,377.14	341.19	0.9	2	198,588.24	2068.63	198,588.24	2068.63
1.0	2	16,103.15	335.48	16,103.15	335.48	1.0	2	198,913.77	2072.02	198,913.77	2072.02
0.3	3	0.00	0.00	0.00	0.00	0.3	3	166,784.20	1737.34	166,784.20	1737.34

## Data Availability

The genotype–environment cell means matrix underlying all analyses presented in this study is openly available in Zenodo at https://doi.org/10.5281/zenodo.20423684. Individual plot data are available from the corresponding author upon reasonable request.

## Abbreviations

The following abbreviations are used in this manuscript:

AMMI.	Additive Main Effects and Multiplicative Interaction.
GGE	Genotype + Genotype–Environment
UAG	Unified AMMI-GGE
UAGI	Unified AMMI-GGE Index
WWW	Which–Won–Where
IPCA	Interaction Principal Component Axis
LOEO	Leave-One-Environment-Out
LOGO	Leave-One-Genotype-Out
LOCO	Leave-One-Combination-Out
LOO	Two-Way Leave-One-Out
PRESS	Prediction Residual Sum of Squares
CV-MSE	Cross-Validated Mean Square Error

## Appendix A

### Appendix A.1. Environmental Characterization of the Three Growing Seasons

E1 (2021/2022). The 2021/2022 growing season was the most precipitation-abundant of the three environments, with a total of 530.8 mm over the September–July period. Rainfall was well-distributed across all major growth phases, including the winter dormancy period (193.8 mm, November–February), spring growth (95.0 mm, March–April), and grain-filling phase (102.0 mm, May–June). Mean monthly temperatures were moderate throughout, with the coldest month being January (1.5 °C) and the seasonal mean reaching 10.6 °C. The balanced precipitation regime and moderate thermal conditions created favorable and non-limiting growing conditions, making E1 the reference environment for broadly adapted genotypes.

E2 (2022/2023). The 2022/2023 season was the most limiting environment, recording the lowest total precipitation of 389.2 mm. Despite a wet September (93.6 mm), the subsequent months were notably dry, particularly during the critical grain-filling phase (May–June: 37.0 mm, with June recording only 2.8 mm). The winter period was also relatively dry (72.6 mm, November–February) but thermally mild (mean January temperature 5.3 °C, the highest across all seasons). The seasonal mean temperature of 11.5 °C and the pronounced late-season drought stress made E2 the most discriminating environment for genotype stability, imposing substantial reproductive-phase water deficit.

**Table A1:** Monthly precipitation and mean temperature at Dobrudzha Agricultural Institute, General Toshevo, Bulgaria (43°42′N, 28°02′E), across the three growing seasons corresponding to environments E1–E3.

Environment/Year	Sep	Oct	Nov	Dec	Jan	Feb	Mar	Apr	May	Jun	Jul	Total/Mean
Precipitation (mm)												
E1 (2021/2022)	8.0	91.6	42.4	89.2	31.4	30.8	19.0	76.0	25.6	76.4	40.4	530.8
E2 (2022/2023)	93.6	6.0	29.4	28.4	29.0	7.4	38.8	79.0	34.2	2.8	40.6	389.2
E3 (2023/2024)	0.6	66.0	126.4	53.0	20.2	2.0	15.6	107.4	61.0	0.8	12.0	465.0
Mean temperature (°C)												
E1 (2021/2022)	16.8	10.2	8.3	3.5	1.5	3.9	2.5	10.8	15.6	20.2	22.7	10.5
E2 (2022/2023)	17.5	12.9	9.1	4.8	5.3	2.8	6.6	9.5	14.6	20.1	24.1	11.6
E3 (2023/2024)	20.4	16.1	8.8	5.2	1.7	7.3	7.5	14.4	14.1	22.8	25.9	13.1

Note: Total precipitation (mm) and mean temperature (°C) are calculated over the September–July growing season period. E1 = 2021/2022 (total precipitation 530.8 mm); E2 = 2022/2023 (total precipitation 389.2 mm, driest season); E3 = 2023/2024 (total precipitation 465.0 mm). Mean temperatures over the growing season: E1 = 10.6 °C; E2 = 11.5 °C; E3 = 13.1 °C (warmest season).

E3 (2023/2024). The 2023/2024 season was intermediate in total precipitation (465.0 mm) but distinct in its seasonal distribution. An exceptionally wet autumn and winter (November: 126.4 mm; total November-February: 201.6 mm) contrasted with adequate spring conditions (March-April: 123.0 mm) but had reduced grain-filling rainfall (May–June: 61.8 mm) and the driest June across all seasons (0.76 mm). E3 was the warmest environment, with a seasonal mean of 13.1 °C and the highest recorded July temperature (25.9 °C), indicating terminal heat stress during grain maturation.

The marked differences in precipitation distribution and temperature profiles among the three seasons generated the substantial genotype–environment interaction detected in the ANOVA (F = 11.29, *p* < 0.001; Table 1) and provide the meteorological basis for the three-sector mega-environment structure identified by the UAG Which–Won–Where analysis (Section 2.2.4).

### Appendix A.2. Plant Material

**Table A2:** Characteristics of the sixteen triticale genotypes evaluated in the multi-environment trials (2021/2022–2023/2024) at the Dobrudzha Agricultural Institute, General Toshevo, Bulgaria.

Code	Designation	Material Type	Cross Combination	Breeding Method	Year of Registration
Advanced breeding lines (G1–G12)—F_6_–F_7_ pedigree selections, crosses made 2014, Dobrudzha Agricultural Institute					
G1	12/14-18-8-48	Advanced breeding line	Atila × Accord	Pedigree	—
G2	12/14-5-11-63	Advanced breeding line	Atila × Accord	Pedigree	—
G3	24/14-17-10-88	Advanced breeding line	Accord × Irnik	Pedigree	—
G4	24/14-33-п-63	Advanced breeding line	Accord × Irnik	Pedigree	—
G5	26/14-5-15-32	Advanced breeding line	Accord × Dobrudzhanets	Pedigree	—
G6	28/14-7-6-40	Advanced breeding line	Accord × Doni 52	Pedigree	—
G7	28/14-1-13-56	Advanced breeding line	Accord × Doni 52	Pedigree	—
G8	34/14-15-12-33	Advanced breeding line	Respect × Irnik	Pedigree	—
G9	35/14-11-12-51	Advanced breeding line	Respect × Bumerang	Pedigree	—
G10	38/14-20-3-78	Advanced breeding line	Respect × Doni 52	Pedigree	—
G11	38/14-1-п-88	Advanced breeding line	Respect × Doni 52	Pedigree	—
G12	38/14-20-3-83	Advanced breeding line	Respect × Doni 52	Pedigree	—
Local check varieties (A, V, R, K)—registered cultivars used as internal standards					
A	AD-7291	Local check variety	—	—	pre-1989
V	Vihren	Local check variety	—	—	pre-1989
R	Rakita	Local check variety	—	—	pre-1989
K	Kolorit	Local check variety	—	—	2006

Note: All twelve breeding lines (G1–G12) are advanced pedigree selections derived from crosses made in 2014 at the Dobrudzha Agricultural Institute, General Toshevo, Bulgaria. Designation codes follow the institute’s internal numbering system. The four check varieties represent local standards used in Bulgarian triticale breeding programs: AD-7291 (A), Vihren (V), and Rakita (R) are historical standards registered before 1989, serving as long-term reference points for yield and adaptation. Kolorit (K), registered in 2006, represents the current performance benchmark in Bulgarian triticale evaluation. The symbol ‘п’ in breeding line designations denotes a bulk selection generation in the pedigree scheme.
